# Split green fluorescent protein as a tool to study infection with a plant pathogen, *Cauliflower mosaic virus*

**DOI:** 10.1371/journal.pone.0213087

**Published:** 2019-03-06

**Authors:** Beatriz Dáder, Myriam Burckbuchler, Jean-Luc Macia, Carine Alcon, Catherine Curie, Daniel Gargani, Jaclyn S. Zhou, James C. K. Ng, Véronique Brault, Martin Drucker

**Affiliations:** 1 BGPI, INRA Centre Occitanie–Montpellier, SupAgro, CIRAD, Montpellier, France; 2 SVQV, INRA Centre Grand Est—Colmar, Université de Strasbourg, Colmar, France; 3 BPMP, CNRS, INRA Centre Occitanie—Montpellier, SupAgro, Univ Montpellier, Montpellier, France; 4 Department of Microbiology and Plant Pathology, Center for Infectious Diseases and Vector Research, University of California, Riverside, Riverside, CA, United States of America; Stony Brook University, UNITED STATES

## Abstract

The split GFP technique is based on the auto-assembly of GFP when two polypeptides–GFP1-10 (residues 1–214; the detector) and GFP11 (residues 215–230; the tag)–both non-fluorescing on their own, associate spontaneously to form a fluorescent molecule. We evaluated this technique for its efficacy in contributing to the characterization of *Cauliflower mosaic virus* (CaMV) infection. A recombinant CaMV with GFP11 fused to the viral protein P6 (a key player in CaMV infection and major constituent of viral factory inclusions that arise during infection) was constructed and used to inoculate transgenic *Arabidopsis thaliana* expressing GFP1-10. The mutant virus (CaMV_11P6_) was infectious, aphid-transmissible and the insertion was stable over many passages. Symptoms on infected plants were delayed and milder. Viral protein accumulation, especially of recombinant 11P6, was greatly decreased, impeding its detection early in infection. Nonetheless, spread of infection from the inoculated leaf to other leaves was followed by whole plant imaging. Infected cells displayed in real time confocal laser scanning microscopy fluorescence in wild type-looking virus factories. Thus, it allowed for the first time to track a CaMV protein *in vivo* in the context of an authentic infection. 11P6 was immunoprecipitated with anti-GFP nanobodies, presenting a new application for the split GFP system in protein-protein interaction assays and proteomics. Taken together, split GFP can be an attractive alternative to using the entire GFP for protein tagging.

## Introduction

Genetic tagging of proteins with fluorescent proteins has revolutionized cell and protein biology by assisting the study of protein localization and protein interactions in real time and *in situ* from the subcellular to organismal scale. However, fusing an entire fluorescent protein (~25 kD) to a protein of interest may impair correct protein folding or interfere with protein functions [1]. Further, insertion of fluorescent protein encoding sequences (~700 nt) can destabilize genomes, especially small genomes like those of many viruses, resulting in rapid elimination of the recombinant sequence (for example [2,3]). A solution to these undesirable occurrences is to fuse the protein of interest to small (<20 amino acids corresponding to 60 nt) epitope tags that are less prone to compromising protein function and genome stability. Accordingly tagged proteins can then be detected by antibody-based techniques. However, this most often requires fixation and permeabilization of cells prior to application of antibodies and is not adapted to live observation. An alternative is the tetracysteine tag, which allows in vivo fluorescent labeling of proteins after incubation of cells with a specific reactive [4]. Disadvantage of this system is the more or less pronounced toxicity of the employed compounds and an often rather high unspecific background label. A promising epitope tag is the split green fluorescent protein (split GFP), where the 11 β-strands that form the barrel-like GFP fluorophore are separated in a big fragment containing the N-terminal 10 β-strands (GFP1-10) and a small (16 amino acids) fragment containing the C-terminal β-strand [5]. The fragments alone are non fluorescent, but the reconstituted GFP1-11 fluoresces. Reconstitution is spontaneous, occurs *in vivo* and *in vitro* and does not require any additional factors. Therefore, the split GFP technique cannot be used to detect protein-protein interactions, in contrast to the bimolecular fluorescent complementation assay (BIFC). Advantages of the split GFP technique is the possibility to track proteins when the complete GFP cannot be used, to label proteins exclusively in specific target cells expressing GFP1-10 [6,7], and to label proteins in extracts or in fixed cells, as an alternative approach to immunofluorescence [5,8].

**Fig 1:**
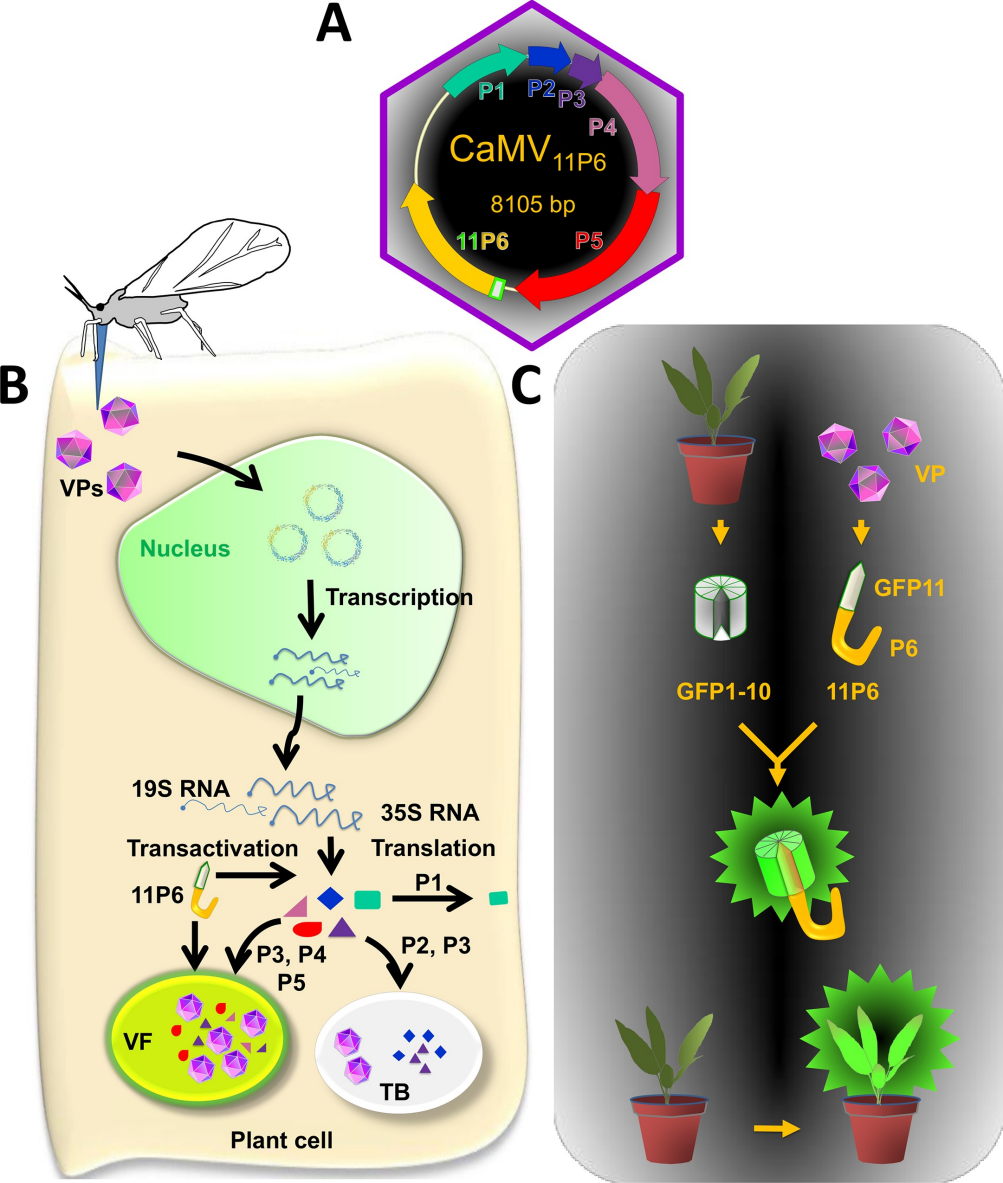
Presentation of the CaMV infection cycle and the split GFP system. (A) The circular double-stranded DNA genome (~8 kbp; circle with arrows) of CaMV is encapsidated in an icosahedral virus particle (mauve hexagon) and codes for six proteins (P1-P6, arrows) that are detected in infected plants. The GFP11 tag (grey box with green border) is fused to the P6 coding sequence yielding 11P6. (B) The infection cycle starts with virus particles (VPs) being delivered into the cytoplasm of a plant cell after it has been punctured by the stylets of an aphid vector. VPs dock at the nuclear envelope and disassemble to allow the naked viral DNA to enter the nucleus. There, the viral genome is transcribed to produce two mRNAs, the 19S RNA encoding P6, and the pregenomic, polycistronic 35S RNA encoding also the other viral proteins. P6 belongs to the early proteins that are translated in the cytoplasm (note that P6 has been replaced by 11P6 in this study). Within the cytoplasm, P6 accumulates in foci that will give rise to the virus factories [here is exemplified one (VF)] with P6 forming the matrix protein, where all viral synthesis occurs and most progeny VPs are stored. Viral synthesis in the VFs involves many coordinated events including the P6-mediated translation transactivation required for the translation of all viral proteins from the polycistronic 35S RNA. The translation products include P1 or MP, the movement protein that associates with the plasmodesmata and is required for cell-to-cell and systemic movement of the virus; P2 or ATF, the aphid transmission factor that binds the virus particles to the aphid vector mouthparts during plant-to-plant transmission; P3 or VAP, the virus-associated protein, P4 or CP (capsid protein), and P5 or RT, the reverse transcriptase generating progeny DNA genomes from the 35S RNA. P6 or TAV (transactivator-viroplasmin) is, besides a transactivator and VF matrix protein, an RNA silencing suppressor that interferes with specific anti-viral defense pathways. Because CaMV engineered to express 11P6 is infectious (as demonstrated in this study), 11P6 is presumed to be functional in all the above stated P6 activities. Besides VFs, a second type of viral inclusions, the transmission bodies (TBs), forms during infection. TBs contain P2, P3 and some VPs and are entirely dedicated to aphid transmission. (C) The split GFP system used in this study. The transgenic reporter plant (top left) expresses the non-fluorescent GFP1-10 (gray barrel with green outline). When infected with CaMV_11P6_, 11P6 produced during infection associates with GFP1-10, yielding fluorescent GFP1-10/11P6 complexes (green barrel) that can be observed by real time fluorescence microscopy or macroscopy. The aphid drawing in (A) is from [24].

*Cauliflower mosaic virus* (CaMV) is a plant virus with a small circular DNA genome of 8 kb that does not tolerate genome insertions longer than a few hundred nucleotides [9,10], presumably due to size constraints during genome packaging into the restricted volume of the icosahedral virus particle (virion). To overcome these limitations, we adapted the split GFP technique to follow the CaMV protein P6 in the context of an authentic viral infection. P6 (also named transactivator-viroplasmin or TAV) is a key player in the viral replication cycle (see Fig 1 for an overview of the virus and the experimental system) by acting as the matrix protein of the cytoplasmic virus factories (VFs), by serving as a transactivator of viral protein translation, by interacting with host defenses and being the main determinant of disease symptoms (reviewed in [11,12]). P6 is also among the first viral proteins to be expressed during CaMV infection [13] and might thus be an interesting candidate for studies on early infection events.

## Results

**Fig 2:**
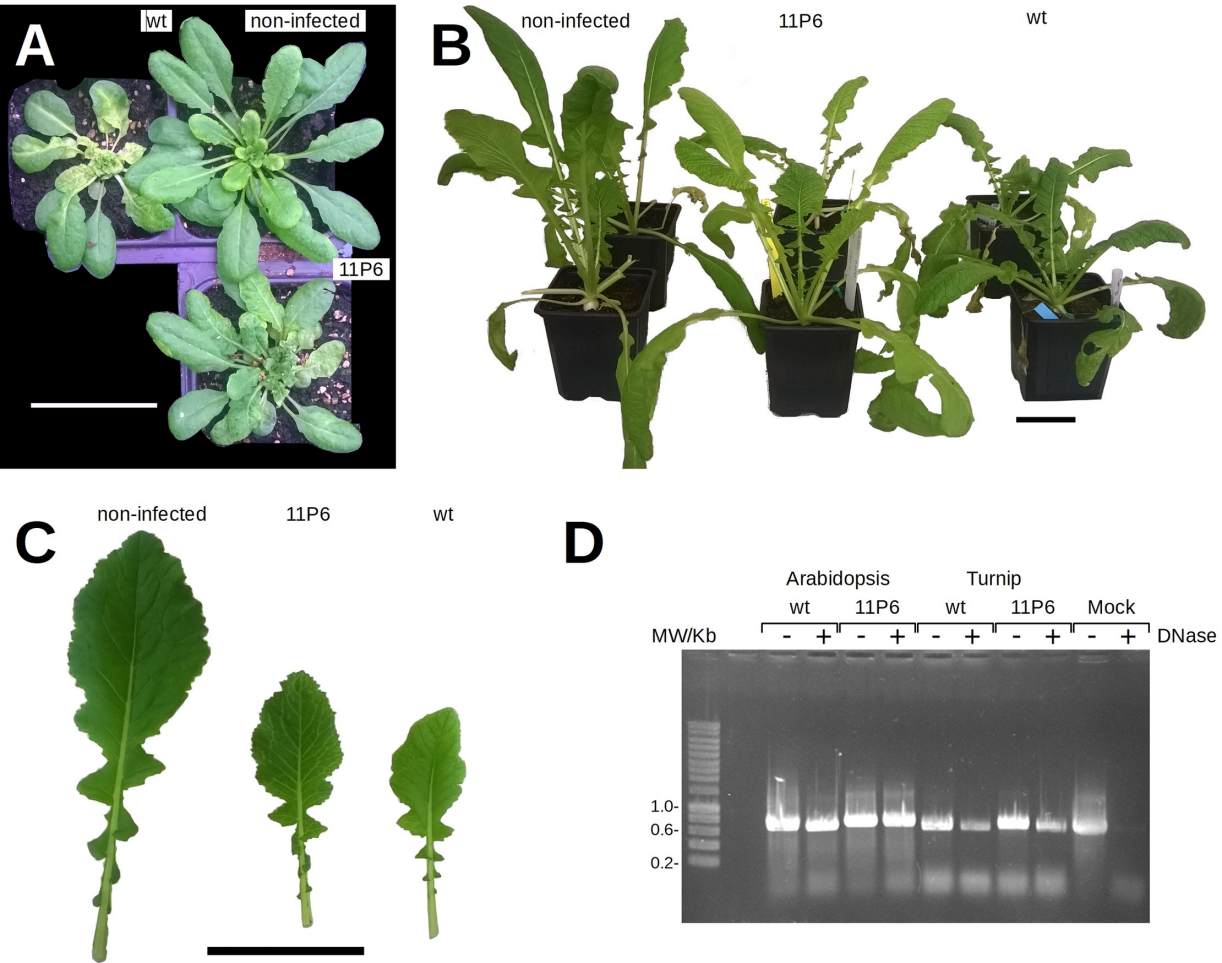
Symptom development on CaMV-infected plants and stability of the insertion. (A) *Arabidopsis thaliana GFP1-10* plants 31 days and (B) turnip plants 36 days after mechanical inoculation with plant extracts prepared from infected (CaMV_wt_ or CaMV_11P6_) or non-infected plants as indicated. Scale bars are 5 cm. (C) Close-up showing attenuated symptoms of turnip leaves of CaMV_11P6_-infected plants compared to CaMV_wt_-infected plants at 27 dpi. For comparison, leaf tissue from a non-infected plant is shown as well. (D) The genomic region encompassing the 11GFP tag was amplified by PCR from total extracts prepared from plants infected with CaMV_wt_ or CaMV_11P6_ as indicated. The viruses had been passaged serially several times (>10 times for *A*. *thaliana* and >4 times for turnip) before the experiment. To determine whether the viral DNA was encapsidated, extracts were (+) or were not (-) incubated with DNase before PCR to digest free DNA. To verify efficiency of the DNase treatment, extracts from mock-inoculated turnip leaf were spiked with CaMV encoding plasmid DNA before DNase treatment (Mock). Amplification of CaMV_wt_ DNA yielded a 655 bp product, amplification of CaMV_11P6_ DNA a 721 bp product.

Site-directed mutagenesis was used to add the GFP11 tag and an amino acid spacer [7] to the N- or C-terminus of P6 in the viral genome of the wild type CaMV strain Cabb B-JI (CaMV_wt_, Fig 1). Despite several attempts, no plasmid containing the C-terminal GFP11 fusion was obtained. Tagging GFP11 to the N-terminus of P6 was successful and the recombinant plasmid, named CaMV_11P6_, was used for mechanical inoculation of transgenic *Arabidopsis thaliana* plants expressing GFP1-10 [7], or of wild type turnip (*Brassica rapa*) plants. Plasmid-inoculated plants developed symptoms (not shown) and were used as virus source for mechanical inoculation of test plants for all further experiments. As Fig 2 shows, turnip and *A*. *thaliana GFP1-10* plants inoculated with leaf extracts developed typical mosaic, yellowing and stunting symptoms like control plants inoculated with CaMV_wt_. However, symptoms were delayed, compared to wild type infection, and appeared in turnips at ~14 and ~21 days post inoculation (dpi) and at ~21 and ~28 dpi (*GFP1-10*), respectively. Symptoms were attenuated in both host plant species inoculated with CaMV_11P6_ (Fig 2). Comparing symptoms in wild type *A*. *thaliana* Col0 or *GFP1-10* plants inoculated with CaMV_wt_ or CaMV_11P6_ did not reveal any differences, suggesting that the transgene did not interfere with infection (S1 Fig). Thus, the results indicated that the mutant virus was infectious and able to accomplish all steps of the in-plant infection cycle (transcription, translation, replication, encapsidation, cell-to-cell movement and systemic movement).

To explore whether CaMV_11P6_ was transmissible from plant-to-plant, we compared aphid transmission of wt and recombinant viruses using infected turnip or *A*. *thaliana GFP1-10* plants as the sources of virus acquisition. CaMV_11P6_ was transmissible. However, transmission rates were significantly lower for CaMV_11P6_- versus CaMV_wt_-infected source plants [turnip: CaMV_wt_ 38±9% vs. CaMV_11P6_ 16±5%; Chi-square test χ^2^ = 13.455, p-value<0.001. *Arabidopsis thaliana GFP1-10*: CaMV_wt_ 22±5% vs. CaMV_11P6_ 8±4%; Chi-square test χ^2^ = 5.260, p-value = 0.022 (Table 1)].

**Table 1:** Aphid transmission rate of CaMV_11P6_.

	Turnip		*A*. *thaliana GFP1-10*	
Infected source plant	wt	11P6	wt	11P6
Infected plants (%)	38±9	16±5	22±5	8±4
Infected/total plants	54/144	15/96	31/144	5/61

Starved aphids were placed on detached infected leaves for acquisition feeding. Following a 5 minutes acquisition access period, a single aphid was transferred to a healthy turnip or *A*. *thaliana GFP1-10* plant for an inoculation access period of 4 h. Transmission rates were recorded 3 weeks later by visual inspection. The data were pooled from three independent aphid transmission experiments. Shown are mean transmission rates ± S.E.

To test the stability of the GFP11 insertion in the viral genome, serial mechanical passages of CaMV_11P6_ were carried out in turnip and *A*. *thaliana GFP1-10* plants. PCR analysis of whole cell extracts indicated that an insertion of the expected size was maintained in the CaMV genome (Fig 2D) for at least 10 serial passages in *A*. *thaliana* plants and for 4 serial passages in turnip plants. Sequencing showed that the insert was the GFP11 tag (see Supplementary Data). However, in one plant we detected a deletion of 6 nucleotides in the spacer region between the GFP11 tag and P6 reducing the spacer from DGGGGS to DGGS. In another plant infected with mechanically passaged CaMV_11P6_, P2 became undetectable by Western blot. PCR analysis indicated a deletion in the P2 ORF (S2 Fig). Stability of CaMV_11P6_ particles, as judged by DNase protection assays (Fig 2D, S3 Fig), seemed to be slightly affected in turnip but not in *A*. *thaliana*. We have no explanation for this.

**Fig 3:**
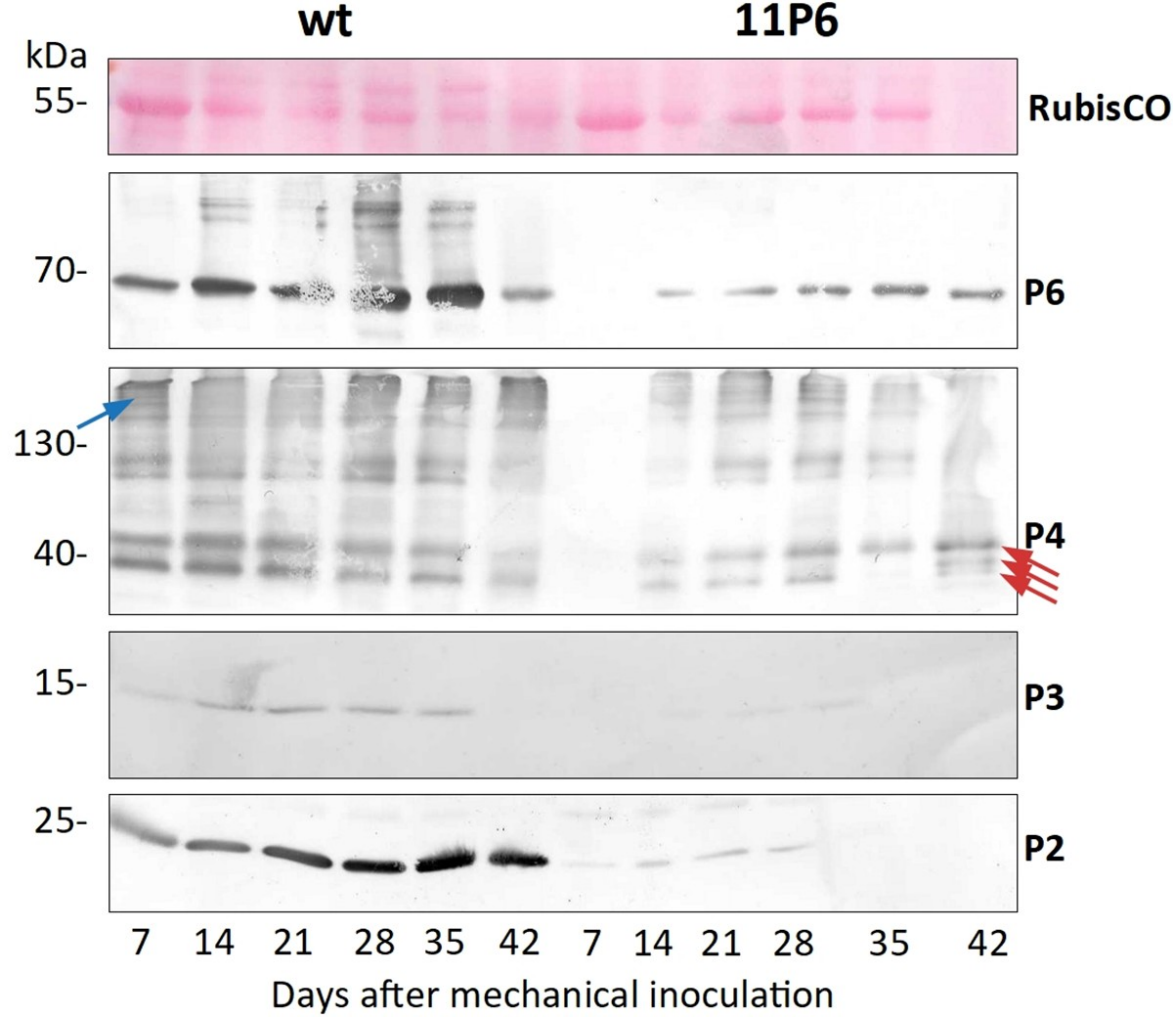
Kinetics of viral protein accumulation in turnip leaves. Total extracts of samples taken from the same systemically infected leaf at the time points indicated were analyzed by Western blotting with antisera against P2, P3, P4 or P6 (indicated by red arrows). The first panel (RuBisCO) shows a loading control (Ponceau Red staining of the large chain RuBisCO subunit). It should be noted that under the electrophoresis conditions used, much of the capsid protein P4 did not enter the gel properly and was retained in the upper part of the gels (blue arrow). The three red arrows in the anti-P4 blot point to the various mature P4 forms detected in infected plants [25]. Shown are the representative Western blots from one experiment out of seven performed.

To better characterize CaMV_11P6_ infection, we analyzed the accumulation of P6, P4 (capsid protein), P3 (virus-associated protein and transmission body component) and P2 (aphid transmission factor) in systemically infected turnip leaves over time. All four proteins were detected at 14 dpi and 28 dpi in plants infected with CaMV_wt_ and CaMV_11P6_, respectively (Fig 3). Accumulation of viral proteins was considerably lower in CaMV_11P6_-infected plants. Quantification using the ImageJ gel analysis macro indicated that the accumulation levels of P6, P4, P3 and P2 in CaMV_11P6_-infected plants were about 5.6±2.1% (n = 6), 28.0±8.0% (n = 2), 9.6% (n = 1; P3 was below the detection limit in most CaMV_11P6_-infected plants) and 19.1±11.6% (n = 6), respectively, of those of P6, P4, P3 and P2 in CaMV_wt_-infected plants. Taken together, CaMV_11P6_ was infectious and caused like CaMV_wt_ systemic infection in the two plant species tested, but symptom development was slower and symptoms were attenuated, concomitant with lower accumulation of viral proteins.

**Fig 4:**
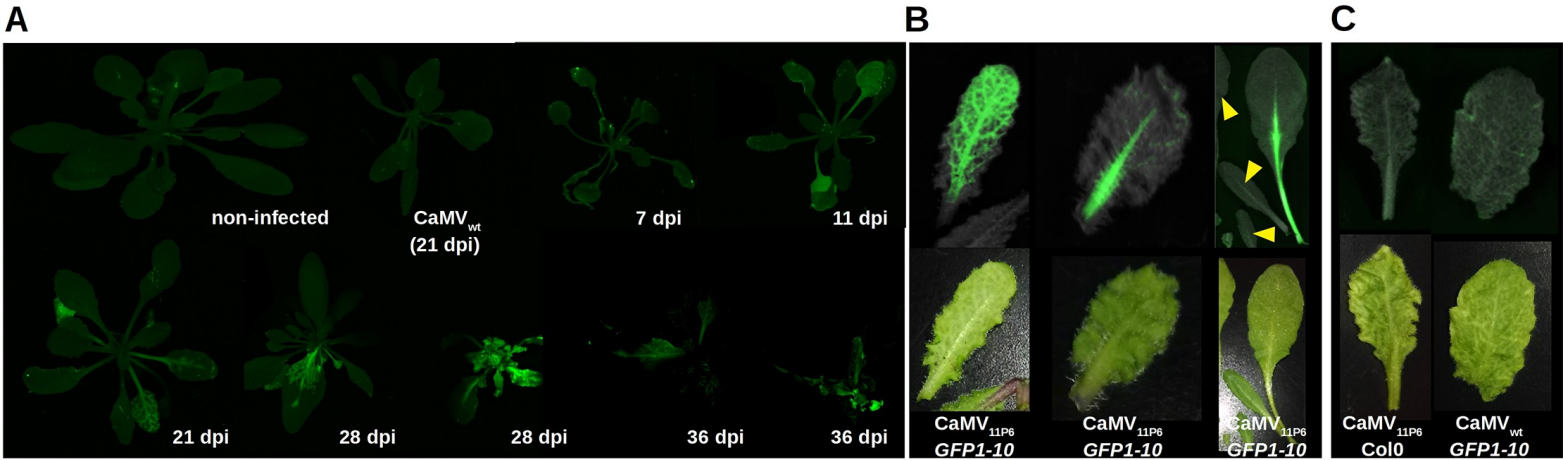
Visualization of 11P6 in CaMV_11P6_-infected *A*. *thaliana GFP1-10* plants. (A) *GFP1-10* plants were mechanically inoculated at different times with CaMV_11P6_ and analyzed at the indicated day post inoculation (dpi) for GFP fluorescence with a fluorescence scanner. The figure is a collage from different acquisitions and plants are presented at different magnification scales. Two plants are presented for 28 dpi and 36 dpi to show different infection states. Non-infected and CaMV_wt_-inoculated (21 dpi) plants are included as negative controls. (B) 11P6 fluorescence is observed before appearance of visual symptoms. *Arabidopsis thaliana GFP1-10* plants were mechanically inoculated with CaMV_11P6_ and leaves analyzed at 24 dpi for GFP fluorescence and visual symptoms with a G:Box (upper panel). The leaves were also analyzed for symptoms with a color camera (lower panel). The three images present 11P6 fluorescence in symptomatic and unsymptomatic leaves. The various leaves in the image to the right are from the same plant. Note that the leaves indicated by the yellow arrowheads are not yet infected. (C) The two pictures show negative controls where either CaMV_11P6_ was inoculated in Col0 plants or CaMV_wt_ in *GFP1-10* plants as indicated. Images were acquired at 32 dpi with a G:Box or a color camera as described above.

Next, we tested whether infection with CaMV_11P6_ could be followed by visualization of GFP fluorescence. For this, CaMV_11P6_-infected *A*. *thaliana GFP1-10* plants were analyzed by whole plant imaging with a fluorescence scanner or a gel documentation system equipped for fluorescence acquisition. Plants displayed green fluorescence, indicating successful reconstitution of split GFP. Fluorescence was visible from 11 dpi and about 21 dpi onwards in inoculated and systemically infected leaves, respectively (Fig 4A). Veins in the leaves were the first to become infected, as evidenced by the onset of GFP fluorescence. Subsequently, GFP fluorescence was observed in the interstitial fields, and continued on until entire leaves were eventually filled with fluorescence (Fig 4A from 21 to 28 days). The fluorescence pattern mirrored the vein bleaching observed in daylight on CaMV_11P6_-infected leaves (Fig 4B). However, the fluorescence was visible in the main veins before the leaves developed symptoms (Fig 4B). *GFP1-10* plants infected with CaMV_wt_ and Col0 plants infected with CaMV_11P6_ did not display fluorescence, demonstrating that the fluorescence was solely due to reconstituted GFP1-10/11P6 complexes (Fig 4C).

**Fig 5:**
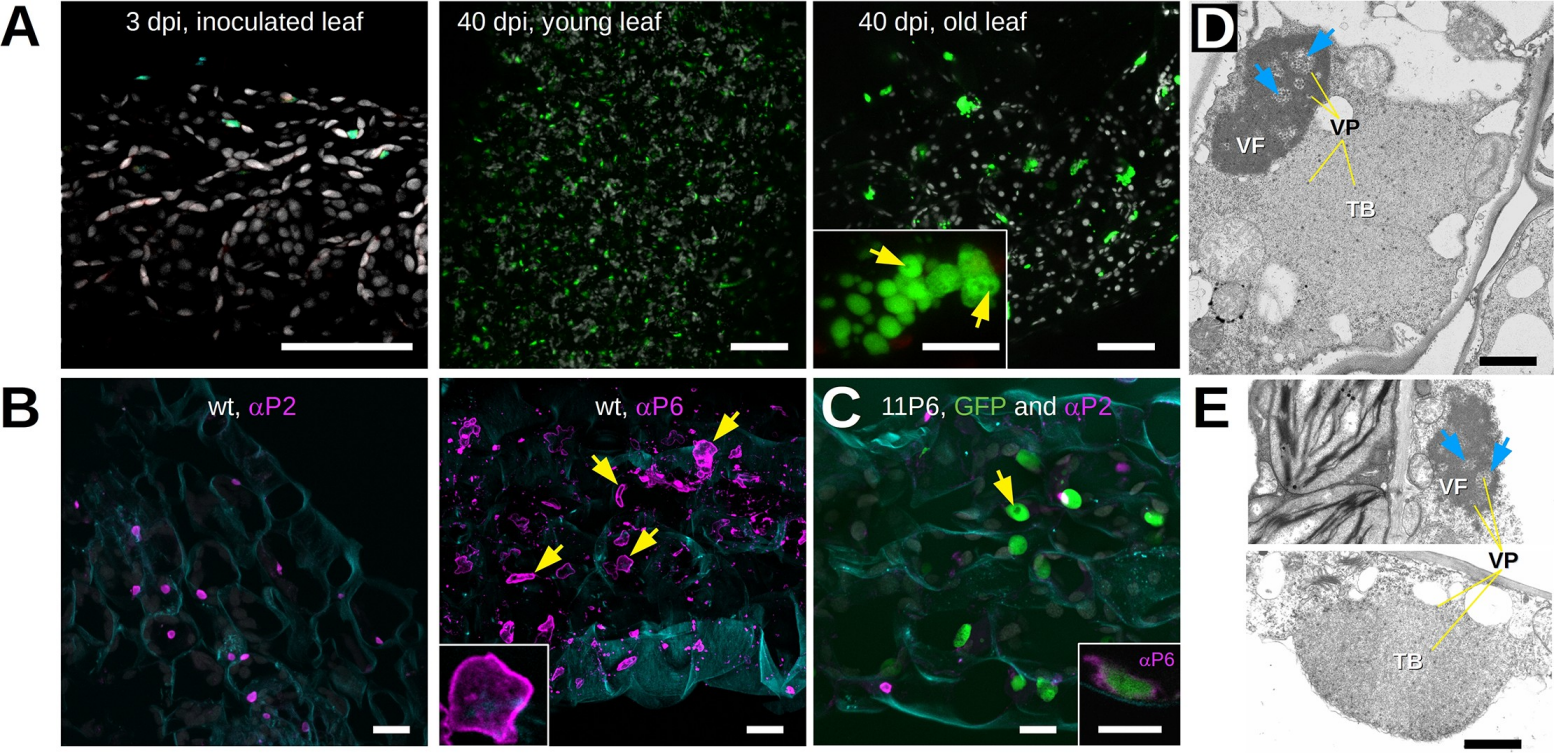
Microscopic analysis of CaMV_11P6_-infected *A*. *thaliana*. (A) *Arabidopsis thaliana GFP1-10* leaves were analyzed by confocal fluorescence microscopy at 3 and 40 days after inoculation (dpi) with CaMV_11P6_. GFP fluorescence and chloroplast autofluorescence are presented in green and grey, respectively. The greenish spots in the leaf tissue at 3 dpi are not due to GFP since they also fluoresced in blue when excited at 405 nm. The inset in the third panel shows details of an 11P6 inclusion in which darker circular spots are visible (yellow arrows). (B) CaMV_wt_-infected tissue sections were immunolabeled (magenta) at 28 dpi using the antisera (αP2 or αP6) as indicated. The yellow arrows point to the stronger stained cortex of immunostained VFs, the inset presents details of a VF. (C) CaMV_11P6_-infected tissue was immunolabeled at 40 dpi using P2 antiserum (magenta) and 11P6 was visualized by fluorescence of the reconstituted split GFP (green). The inset shows a confocal single section of such a green fluorescing inclusion labeled with P6 antiserum (magenta). Note that only the cortex of the inclusion is labeled. The yellow arrows point to putative VF lacunae. Cell walls in (B) and (C) were stained with Fluorescent Brightener 28 and are presented in blue. All confocal images are maximum projections except where indicated. (D) and (E) Transmission electron micrographs presenting (D) a CaMV_wt_ and (E) a CaMV_11P6_-infected cell, both fixed at 33 dpi. VF and TB designate virus factories and transmission bodies, respectively; yellow lines (labeled “VP”) and blue arrows point to virus particles and lacunae, respectively. Different microscopes were used for image acquisition in (D) and (E). Brightness and contrast were corrected to allow better comparison of the micrographs. Scale bars in (A) 50 μm for the overviews and 5 μm for the inset, in (B) and (C) 10 μm and in (D) and (E) 1 μm.

We then used confocal laser scanning fluorescence microscopy to determine the intracellular distribution of 11P6 in infected tissues of *A*. *thaliana GFP1-10* plants. No GFP signal was detected in the inoculated leaves at 3 dpi (Fig 5A). In systemically infected leaves, many fluorescent inclusions of various sizes and shapes were visible (Fig 5A, 40 dpi). Since P6 is the major component of the viral factories (VFs), the fluorescent dots were presumably VFs. The sizes and abundance of the inclusions were dependent on the infection status, being more numerous, but smaller, in younger, newly infected leaves, and less numerous, but larger, in older infected leaves (Fig 5A, 40 dpi). Upon closer observation, small spherical cavities void of fluorescence were detected in the lumen of many inclusions (Fig 5A, 40 dpi old leaf). Tissues infected with CaMV_11P6_ or CaMV_wt_ were also analyzed by immunofluorescence using antibodies raised against P2 and P6 (Fig 5B and 5C). P2-specific label was localized in typical transmission bodies (TBs) in CaMV_wt_ and CaMV_11P6_-infected cells, indicating that 11P6 did not interfere with TB formation. Immunolabeling of P6 revealed numerous irregularly shaped VFs in CaMV_wt_-infected cells. Curiously, the antibody labeled mostly the cortex of VFs but hardly their interior and the small spherical dark spots in the lumen of 11P6 inclusions were not revealed in P6-labeled wt VFs (Fig 5B). Compared to wt VFs, the CaMV_11P6_ inclusions were on average smaller and had a more regular, rounded or multilobular, shape (see inset in Fig 5A). We did P6 immunolabeling of the fluorescent inclusions to identify them as VFs. Anti-P6 labeled predominantly the cortex of the inclusions (inset of Fig 5C). This resembled the P6 label of wt VFs and identified the 11P6 inclusions as VFs. The fact that anti-P6 labeled mainly the cortices of both wt and 11P6 VFs, indicated that the antibody penetrated only badly into the VF matrix, known to be composed principally of P6 [12]. The barely above background immunofluorescence of P6 in the VF lumen might also explain why we could not detect the VF cavities revealed by GFP fluorescence in 11P6 VFs.

To definitely rule out modification of viral inclusions by CaMV_11P6_, we further examined the ultrastructure of VFs and TBs by transmission electron microscopy (TEM). Typical amorphous VFs were detected in cells regardless of whether they were infected with CaMV_wt_ or CaMV_11P6_ (Fig 5D and 5E). The VFs were composed of an electron-dense matrix with many virions embedded within them and also in spherical lacunae, and no structural modifications were noticed in CaMV_11P6_-induced VFs. Our examination of TBs also did not reveal any differences between those produced in CaMV_wt_-infected and in CaMV_11P6_-infected cells. In both cases, typical TBs, characterized by an electron-lucent matrix with a few embedded virions, were detected. Taken together, these observations indicated that TB and VF ultrastructure was not modified by the mutant P6, although on the light microscopic level 11P6 VFs seemed to be smaller and more regularly shaped than wt VFs.

**Fig 6:**
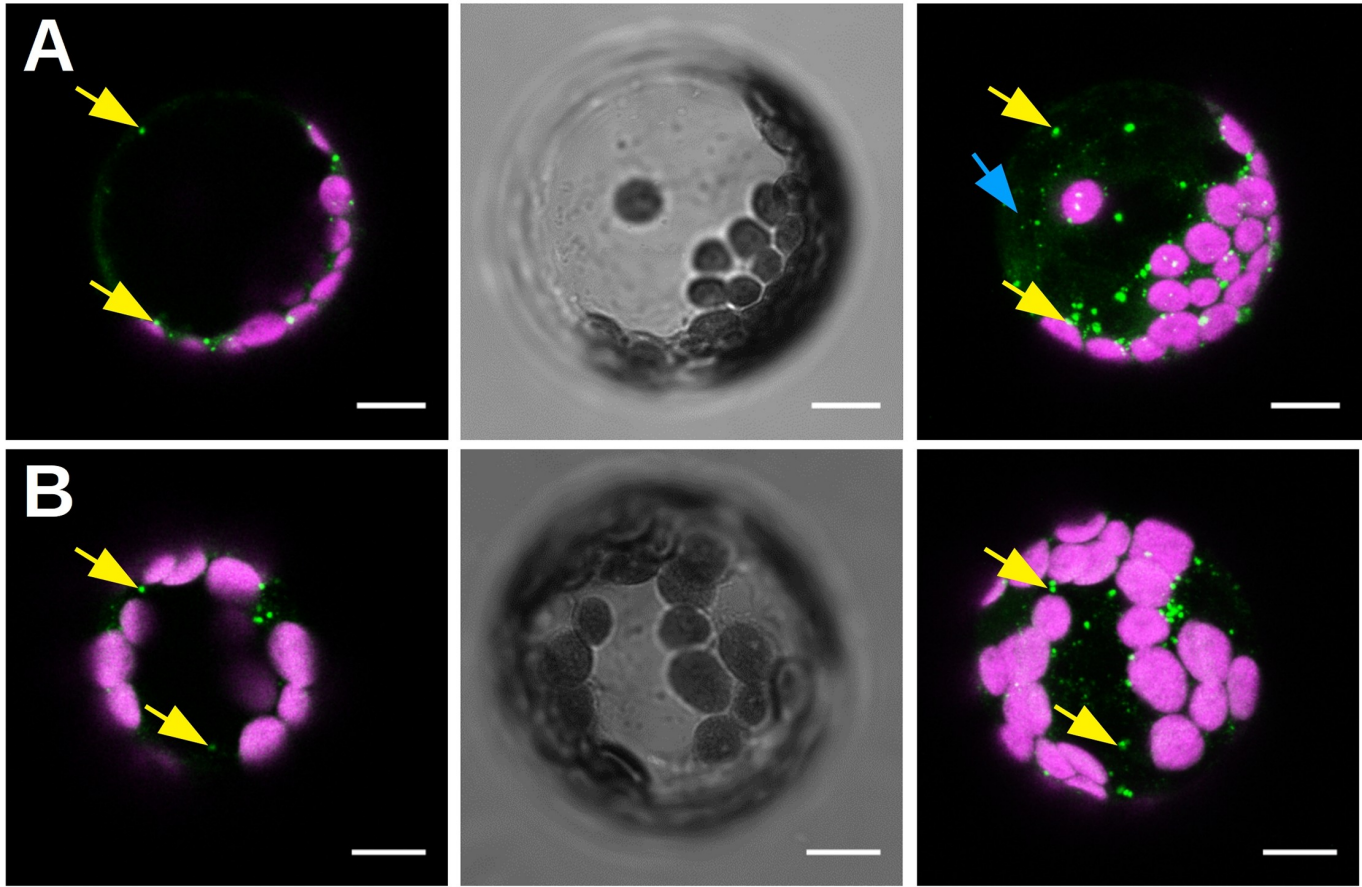
Observation of CaMV_11P6_-transfected *A*. *thaliana GFP1-10* protoplasts. Protoplasts were transfected with infectious CaMV_11P6_ plasmid (A) or with CaMV_11P6_ virus particles (B), and observed 15 h later by confocal fluorescence microscopy. Protoplasts display fluorescent 11P6 foci (green spots indicated by yellow arrows) and weak cytosolic 11P6 label (blue arrow). The images shown in (A) or (B) are from the same protoplast. The left panels present optical single sections, the right panels maximum projections, and the middle panels bright field illumination images. Chloroplasts are presented in magenta. Scale bars in all panels represent 10 μm.

The above results suggested that the CaMV_11P6_/GFP1-10 reporter system might be used to detect early infection events, for example after aphid transmission. However, despite several attempts to identify infection foci in aphid-inoculated leaves, we were unable to detect any 11P6-associated GFP fluorescence emerging close to aphid stylets puncture sites. Therefore, we switched to using protoplast transfection, which allowed us to “inoculate” and monitor a much higher number of cells than would be possible with aphid inoculation. Protoplasts prepared from healthy *A*. *thaliana GFP1-10* leaves were transfected either with a plasmid encoding the CaMV_11P6_ genome or with purified CaMV_11P6_ virus particles, prepared from infected plants. Fifteen hours after inoculation, protoplasts transfected with CaMV_11P6_ plasmid or virus particles displayed weak cytosolic 11P6/GFP1-10 fluorescence, but most fluorescence accumulated in cytosolic aggregates (Fig 6).

**Fig 7:**
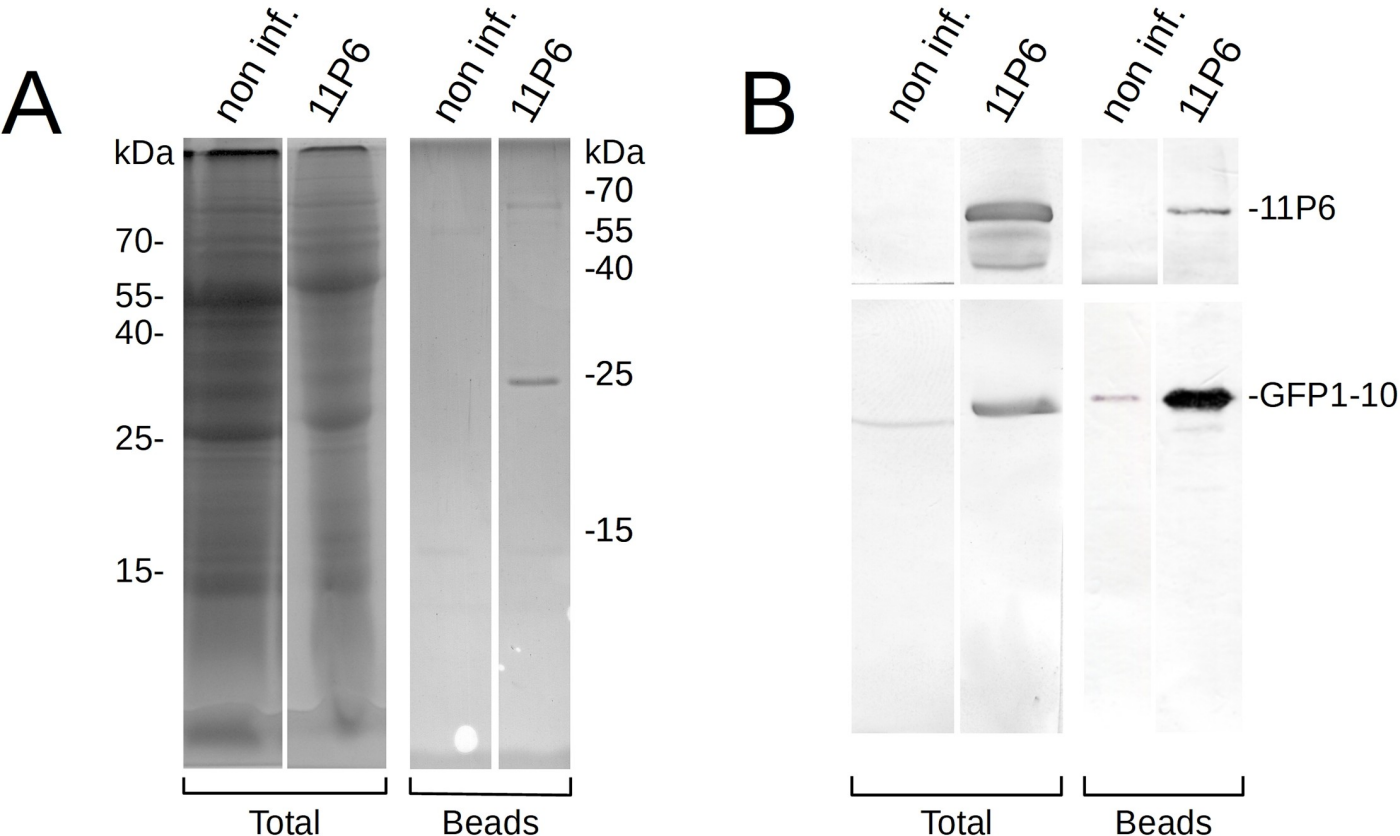
Immunoprecipitation of 11P6. Cell lysates, prepared from non-infected controls (non-inf.) or CaMV_11P6_-infected *A*. *thaliana GFP1-10* plants (11P6), were incubated with magnetic beads with immobilized anti-GFP nanobodies. Total cell extracts (Total) or proteins retained on the beads (Beads) were separated on different gels by SDS-PAGE and analyzed by Coomassie Blue staining or Western blotting. (A) Coomassie blue staining reveals proteins with molecular masses of approximately 65 kD and 25 kD from CaMV_11P6_-infected but not from healthy control lysate. The weakly stained 55 kD and 15 kD proteins visible in the healthy control probably represent carried-over small and large chain RuBisCO subunits, respectively. (B) Membranes were cut in two and revealed for P6 (upper blots) or GFP (lower blots). This allowed to identify the 65 kD protein as 11P6 and the 25 kD protein as GFP1-10.

To ascertain whether the reconstituted split GFP complex was sufficiently stable to allow immunoprecipitation using anti-GFP nanobodies, cell lysates prepared from healthy and CaMV_11P6_-infected *A*. *thaliana GFP1-10* leaves were incubated with anti-GFP nanobodies immobilized on magnetic beads. Then, proteins bound to the beads were analyzed by SDS-PAGE and Western blotting. We observed that 11P6 was retained on the beads, suggesting that 11P6 had, in association with GFP1-10, bound to the immobilized anti-GFP nanobodies (Fig 7).

## Discussion

In this study, we have incorporated a split GFP tag into P6, a key player in CaMV infection. An addition of 66 nucleotides that corresponded to 16 aa of the GFP tag and a 6 aa spacer (2.4 kD in total) yielded an engineered virus CaMV_11P6_. The stability of the GFP-tagged P6 (11P6) expressed from CaMV_11P6_ was maintained for > 10 generations in *A*. *thaliana GFP1-10* and for at least 4 generations in turnip plants. However, in one case a 6 nucleotides deletion resulting in a shortened amino acid spacer between GFP11 and P6 and in another case a partial deletion of ORF2 were observed after prolonged serial passaging of CaMV_11P6_. Whereas the P2 deletion could be have been due to the mechanical passaging that renders the aphid transmission factor P2 functionless, with similar deletions having been reported in naturally occurring CaMV isolates [14], the deletion in the spacer is probably due to compensation of impaired P6 function. CaMV has been used before to express recombinant proteins [9,10]. For this, ORF II (not required for infection) had to be deleted to obtain stable recombinant viruses, and even then expression of the recombinant proteins decreased over time. Here, however, the insertion of 66 nucleotides did not destabilize the P6 sequence itself although in one case and after prolonged passages the spacer sequence between GFP11 and P6 mutated. This indicates relative stability of the insert. We attribute this to the small size of the insert, which should not or only marginally interfere with genome encapsidation, and to the fact that neither additional open reading frames were introduced nor non coding regions between the open reading frames were elongated, both shown to destabilize the CaMV genome [9]. However, symptoms were delayed and attenuated in both hosts, and 11P6, P4, P3 and P2 accumulations were reduced. Since delayed and attenuated symptoms and lower protein accumulations were observed in both transgenic *GFP1-10* and wild type plants, the effects were unlikely to be caused by the reconstituted GFP, but rather due to the sole addition of GFP11 to P6. How 11P6 lowered accumulation of P2, P3 and P4, remains for the time being unknown. Translation of these proteins from the polycistronic 35S RNA requires the trans-activation function of P6 [15]. Since viral DNA levels were similar in CaMV_wt_ and CaMV_11P6_-infected plants, it is probable that 35S RNA levels were also similar in wt and mutant infected plants, pointing to a direct role of 11P6 in lower translation of P2, P3 and P4. Several scenarios are possible. The lower accumulations were due to lower 11P6 protein levels, the insertion of the GFP11 tag affected transactivation, or GFP11 might physically impede protein translation from the 35S RNA. Further investigation is required to resolve this issue.

Examination of CaMV_11P6_ infected *A*. *thaliana GFP1-10* plants by fluorescence microscopy confirmed successful GFP1-10/11P6 complex formation in inclusions (Fig 5). These inclusions were confirmed to be VFs by electron microscopy that revealed no difference in ultrastructure between wt and 11P6 inclusions. 11P6 VFs were smaller than wt VFs, but their most distinguishing feature was their regular ovoid or multilobular shape, compared to the irregular wt VFs. This again can be taken as evidence that the N-terminus of P6 is involved in P6-P6 interaction and in scaffolding the three-dimensional VF structure.

The N-terminus of P6 has been shown to be important for symptom severity and CaMV virulence. For example, alanine substitution of the EKI motif (aa 11–13 of P6) in the CaMV-TAVm3 mutant has been shown to cause delayed and weaker symptoms in turnip plants [16]. These observations correlated with a drastic decrease in accumulation of P6 and, to a lesser extent, P4, but less so for P2 (Fig 3 and [16]). The lower P6 accumulation might explain the lower virulence of the two mutants, i.e. CaMV-TAVm3 and CaMV_11P6_, since P6 is a major symptom severity determinant. However, there are important differences between these two mutants. 11P6 formed wt TBs and VFs whose ultrastructure was indiscernible from wt VFs, whereas P6-TAVm3 formed only small VF-like inclusions containing some virions but apparently no lacunae [16]. Further, CaMV-TAVm3 did not form TBs. Taken together, we provide evidence that P6 accumulation (or stability) and its role in VF and TB formation are different functions of P6’s N-terminus. Accumulation (or stability) of P6 depends on a wild type N-terminus, which is compromised in both mutants. On the other hand, P6’s implication in inclusion body formation requires specifically the EKI motif, which is compromised only in CaMV-TAVm3.

To assess the suitability of CaMV_11P6_ to facilitate the monitoring of early infection events, 11P6-associated GFP fluorescence was followed in mechanically inoculated *A*. *thaliana GFP1-10* plants and in transfected protoplasts. GFP fluorescence appeared in the inoculated leaves of *A*. *thaliana GFP1-10* plants at approximately 11 dpi and was observed in the systemically infected leaves at around 21 dpi (7 days ahead of the appearance of CaMV_11P6_-induced systemic symptoms i.e. 28 dpi). In accordance with a previous report [17], the infection pattern followed typical source-to-sink transport, most probably via the phloem. Whether CaMV passaged through the roots to invade upper leaves, is highly probable, but we have no direct evidence for this. The onset of systemic symptoms induced by CaMV_11P6_ in *A*. *thaliana GFP1-10* was considerably slower than that induced by CaMV_wt_. Using aphids to inoculate CaMV_11P6_ to *A*. *thaliana GFP1-10* plants, we were unable to detect GFP fluorescence at the inoculation sites, although transmission experiments showed that CaMV_11P6_ was transmissible, albeit at significantly lower transmission rates (Table 1). This indicated that CaMV_11P6_-inoculated cells were present in the plant tissues given access to viruliferous aphids, but we were unable to spot them shortly after virus inoculation. Our results contrast with those obtained in a study in which a GFP-tagged *Cucumber mosaic virus* was detected in cells close to the aphid stylet trajectory [18]. The failure to detect cells inoculated with CaMV_11P6_ was probably due to the twenty-fold lower accumulation of 11P6 (compared to wt infection), which decreased the detection limit by about twenty-fold. It is also possible that the amount of CaMV_11P6_ virions inoculated by a single viruliferous aphid was insufficient to allow reconstituted GFP molecules to go above the level of detection in the initially infected cell(s). In CaMV_11P6_-transfected *A*. *thaliana GFP1-10* protoplasts, 11P6-associated GFP fluorescence was detected in small foci 15 h after transfection, which was comparable to that reported in a previous work where P6 inclusions were observed in CaMV_wt_-transfected protoplasts by immunofluorescent localization, likewise 15 h after transfection [19]. The detectability of the P6 targets is remarkable given the different detection platforms used i.e. GFP fluorescence produced by the reconstitution of split GFP peptides (in the current study) vs. immunofluorescence [19]. With regards to inocula (plasmid vs. virus particles) used for protoplast transfection, no differences were observed in 11P6 accumulation patterns–they all displayed small inclusions as well as some cytoplasmic localization. Cytoplasmic localization of P6 has previously been reported for some ectopically expressed P6 mutants [20], but to our knowledge, this has yet to be observed for P6 expressed in the context of a viral infection. However, since P6 is translated in the cytoplasm and a small fraction of it shuttles between the cytoplasm and the nucleus [20], it is not surprising to detect it in the cytoplasm. The reason that it was not described there before, might be that most work on P6 localization was done at the electron microscopic level, where cytoplasmic P6 might have been overlooked. P6 has previously been described in the nucleus of protoplasts purified from infected plants i.e. in the context of natural infection [20]. We did not detect nuclear 11P6 in CaMV_11P6_-transfected protoplasts. This could be due to accumulation below detection limits, or, alternatively, P6 shuttles to the nucleus later in infection.

To the best of our knowledge, this is the first demonstration of the use of a split GFP system for the immunoprecipitation of a viral or other protein. The GFP1-10:11P6 complex was strong enough to withstand the rather drastic incubation conditions (100 mM EGTA, see Material and Methods), and was pulled down by anti-GFP nanobodies. Therefore, split GFP has the great potential for applications that are aimed at confirming the presence of a known protein or identifying new protein-protein interactions. Interestingly, uncomplemented GFP1-10 appeared to accumulate to much lower levels in healthy *GFP1-10* plants than in *GFP1-10* plants infected with CaMV_11P6_. This observation suggested that GFP1-10 was either not highly expressed in uninfected plants or that it was unstable in the uncomplemented form in *GFP1-10* plants.

Taken together, GFP11 tagging of viral proteins (demonstrated here using CaMV_11P6_) is a useful technique for tracking their movement and analyzing their interactions with host proteins, especially in situations where incorporating the entire GFP coding sequence is not feasible, as is the case with CaMV. Probably for this reason, most *in vivo* characterization of CaMV P6 has been carried out using ectopically expressed GFP-P6 fusions [20–23]. Our approach might open the door to study P6 in real time and in the context of a genuine CaMV infection, even if the low accumulation of 11P6 somewhat limited its suitability. It remains to be seen whether this issue will accompany the GFP11 tagging of other CaMV proteins or the proteins of other viruses that we are currently testing. Nevertheless, we have demonstrated that this technology is clearly suitable for applications involving organisms that cannot accommodate the complete GFP coding sequence in their genomes. It might not only be useful for the study of viruses that do not tolerate bigger genomic insertions, but also ease study of viruses that tolerate bigger insertions but then are not encapsidated. A drawback of tracking proteins live with the split GFP technique is that it requires *in situ* expression of GFP1-10, produced either in transgenic organisms or after cotransfection in transient expression systems. GFP11 tagging should also be applicable in situations where the reassembly of the whole GFP protein renders the protein of interest non-functional or where no GFP1-10 expressing cells or organisms are available. In this case, it is possible to add recombinant purified GFP1-10 protein to cell lysates or fixed cells [8], either to visualize or to immunoprecipitate the tagged protein. Alternatively, GFP1-10 expression can be induced in transgenic cells at a desired time point to allow a GFP11-tagged protein, before visualization, to mature and exert its functions *in vivo* without the possible interference by a massive GFP molecule.

## Materials and methods

### Plants

Turnip [*Brassica rapa* L. cv. ‘Just Right’ (Takii Europe, De Kwakel, The Netherlands)] plants were grown in a greenhouse at 24/15°C day/night and 60% relative humidity with a 14/10 h day/night photoperiod. Transgenic *A*. *thaliana* L. Col 0 expressing GFP1-10 constitutively under control of the 35S promoter [7] were grown in a climate chamber at 20/17°C day/night and 60% relative humidity with a 8/16 h day/night photoperiod. All seeds were planted with Humin-Substrat N2, pH 5.8 (Neuhaus, Geeste, Germany) and watered with a nutrient solution.

### Plasmid construction and inoculation

To construct the infectious plasmid pGreen-35S-CaMV_11P6_ coding for 1.2 genomes (to allow transcription of a full length 35S RNA) of CaMV_11P6_ under control of the 35S promoter, GFP11 (RDHMVLHEYVNAAGIT) and a linker (DGGGGS) were fused to the N-terminus of the P6 protein of CaMV strain B-JI [26] in the plasmid pGreen 35S B-JI [19], using the Q5 site-directed mutagenesis PCR cloning kit (New England Biolabs, Evry, France). The presence and identity of the insert in the plasmid and in infected plants was verified by Sanger sequencing.

Two-week-old turnips and four-week-old *A*. *thaliana*, depending on the experiment, were mechanically inoculated either with plasmids or with plant extracts that were prepared by grinding infected leaves in 10 mM HEPES buffer pH 8.0. To facilitate penetration, carborundum was added to the inoculum or the leaf cuticle was previously rubbed with this abrasive.

### Aphid transmission tests

Non-viruliferous *Myzus persicae* Sulzer aphids were reared on healthy *Solanum melongena* L. in a growth chamber at 24/19°C day/night and 60% relative humidity with a 14/10 h day/night photoperiod. Aphids were starved for 1 h at room temperature and transferred to detached virus-infected leaves for a 5 min acquisition access period (AAP). Afterwards, one single feeding aphid was transferred onto each turnip or *A*. *thaliana GFP1-10* test plant for an inoculation access period of 4 h (IAP). Then they were sprayed with insecticide Pirimor (Syngenta, Saint-Sauveur, France) at a concentration of 1 g L^-1^ and placed in a growth chamber at 23/15°C day/night and 60% relative humidity with a 12/12 h day/night photoperiod for 3 weeks for visual inspection of symptom development.

### Kinetics of viral protein accumulation

To determine the accumulation of viral proteins in infected turnip plants, we collected leaf samples from plants mechanically inoculated with sap prepared from CaMV_wt_-infected, CaMV_11P6_-infected or mock-inoculated plants weekly over a 42 days post-inoculation (dpi) period. Supernatant containing total proteins were prepared by grinding a leaf disk (0.5 cm diameter) in 0.2 ml extraction buffer (10 mM HEPES, pH 8) and centrifugation at 13,600 g for 10 min at 4°C. Supernatants were mixed with an appropriate volume of 4x or 6x Laemmli buffer [27], heated for 5 min at 95°C, loaded in duplicates on two 13.5% polyacrylamide gels and subjected to SDS-PAGE according to [27]. Separated proteins were transferred onto nitrocellulose membranes that were cut in two. The upper parts were incubated with P4 antibody [28] or P6 antiserum [29], and the lower parts with P2 antiserum [30], P3 antiserum [31] or monoclonal GFP antibody (Chromotek, Planegg-Martinsried, Germany), followed by incubation with alkaline phosphatase-conjugated or peroxidase-conjugated secondary antibodies and chromogenic detection with the NBT/BCIP substrate or by ECL.

### Immunofluorescence of leaf sections

Eight weeks old *A*. *thaliana GFP1-10* leaves infected with CaMV_wt_ or CaMV_11P6_ were cut into 0.5 cm^2^-squares and fixed at room temperature for 90 min with 1% glutaraldehyde (v/v) in 50 mM HEPES, pH 7. After three rinses with 1 x phosphate buffered saline (150 mM NaCl, 50 mM NaPO_4_, pH 7.4), leaf pieces were embedded in 5% low-melting point agarose in water, and cut into 50 μm sections using a HM 650 V vibratome (Thermo Scientific, Villebon-sur-Yvette, France). Sections were collected, transferred to cell culture plates, incubated for 1 h in 0.1% NaBH_4_ and blocked with 3% BSA in Tris-buffered saline (TBS, 150 mM NaCl, 50 mM Tris, pH 7.4) for 90 min. Sections were then incubated overnight with rabbit anti-P2 or rat anti-P6 primary antisera at a 1:100 dilution in 5% BSA in TBS at 4°C. After three rinses with TBS, sections were incubated for 6 h at room temperature with Alexa Fluor conjugates (Thermo Scientific, Villebon-sur-Yvette, France) at a 1:100 dilution. After rinsing three times with TBS, cell walls were stained with 0.002% Fluorescent Brightener 28 (Sigma Aldrich, Saint-Quentin-Fallavier, France) in TBS, and washed another two times with TBS. Sections were mounted in FluoroShield medium (Sigma Aldrich, Saint-Quentin-Fallavier, France) on glass microscope slides. For transmission electron microscopy, *A*. *thaliana* leaves were fixed with 4% glutaraldehyde, postfixed with 2% OsO_4_, and embedded in Epon resin as previously described [32].

### Protoplast transfection

Protoplast isolation and PEG/calcium-mediated transfection of 4 weeks old *A*. *thaliana GFP1-10* seedlings were as previously described [33]. 10 μg of plasmid DNA or virus particles, purified as previously described [34], were used to transfect 2*10^4^ protoplasts. For this, 100 μl of protoplasts in W5 buffer (154 mM NaCl, 125 mM CaCl_2_, 5 mM KCl, 5 mM glucose, 2 mM MES pH 5.7) were gently mixed with 10 μl plasmid or virus particles. 110 μl PEG buffer (40% PEG 4000, 0.2 M mannitol, 0.1 M CaCl_2_) was added carefully to the protoplasts and incubated for 15 min at room temperature. The PEG/protoplast suspension was diluted stepwise with 1.8 ml of W5 buffer and centrifuged for 5 min at 100 g. This washing step was repeated before resuspending the protoplasts in 100 μl of MMG buffer (0.4 M mannitol, 15 mM MgCl_2_, 4 mM MES). After addition of 900 μl W1 buffer (0.5 M mannitol, 20 mM Kcl, 4 mM MES pH 5.7), the protoplasts were incubated in a multiwell cell culture plate in darkness at 21°C.

### Image acquisition

A Typhoon FLA 9000 scanner (GE Healthcare Life Sciences, Velizy-Villacoublay, France) was used to record the GFP fluorescence in whole leaves. GFP was excited with a 473 nm laser and fluorescence was collected after passage through a 530±20 nm band pass filter. Pixel size was set to 10–500 μm, depending on the experiment. Alternatively, plants were placed in a G:Box F2 gel documentation system (Syngene, VWR France, Fontenay-sous-Bois, France). GFP fluorescence was recorded after excitation with the blue LED module and passage of the fluorescence through a 525±15 nm band pass filter. Grey scale bright field images were acquired with white illumination.

To visualize symptoms, leaves or plants were photographed with a smartphone color camera (Nokia, Boulogne-Billancourt, France).

Tissue sections were observed with a Zeiss LSM700 confocal microscope (Carl Zeiss, Marly le Roi, France) operated in sequential mode and using a 20x air or 63x oil immersion objective. Fluorescent Brightener 28 was excited with the 405 nm LED laser and emission was collected from 405–530 nm; GFP was excited with the 488 nm laser and the mirror and long pass filter set were used to collect GFP fluorescence (from 490–530 nm) and chlorophyll autofluorescence (from 560–700 nm); Alexa Fluor 594 was excited with a 555 nm laser, and the mirror was set to record emission from 555–620 nm. Raw images were processed using ZEN software and the final figures were prepared using the Zeiss LSM or ImageJ softwares. Confocal imaging of protoplasts was performed using a Leica TCS SP8 laser scanning microscope with a 40x water N.A. 1.1 immersion objective (Leica Microsystemes, Nanterre, France). GFP and chlorophyll autofluorescence were collected using the line sequential scanning mode between 500–540 nm and 680–700 nm, respectively, after excitation at 488 nm (with 5% and 0.8% of laser power, respectively). Images were processed using the ImageJ software. Electron microscopy was performed using a Jeol JEM 100CX II or a JEM-1400Flash microscope (JEOL Europe, Croissy sur Seine, France) operated at 60–80 kV.

Brightness and contrast of images was adjusted, the same settings were applied to the whole images.

### Immunoprecipitation of GFP11-fusion protein

*Arabidopsis thaliana GFP1-10* leaves were frozen, ground and resuspended in 500 μl ice-cold SES buffer (200 mM Tris, pH 7.6, 100 mM EGTA, 50 mM MgCl_2_) with 1% Tween20. Samples were incubated on ice for 30 min, centrifugated at 20,000 g for 10 min, and the lysate was diluted in another 300 μl of SES buffer. GFP-Trap agarose magnetic beads (ChromoTek, Planegg-Martinsried, Germany) were equilibrated in SES buffer and the lysate was incubated with the beads for 60 minutes at 4°C to magnetically pull down GFP-associated proteins and potential plant partners. Proteins were eluted by boiling in Laemmli buffer for 10 minutes at 95°C. Classic 13.5% SDS-PAGE was performed as described above, and proteins were analyzed by Western blotting as indicated above or by Coomassie Blue staining.

### DNA analysis

Leaf disks were obtained by punching leaves with the lid of a 1.5 ml reaction tube. 100 μl of 10 mM HEPES buffer pH 8.0 were added per leaf disk and the tissue was homogenized manually with a plastic pistil or, after adding 2 glass beads, with a 30 s stroke in a Retsch MM 2000 mixer mill (Retsch France, Eragny sur Oise, France). For DNase protection assays, the samples were diluted tenfold and 1 μl DNase buffer and 1 μl RQ1 DNase (Promega, Charbonnières-les-Bains, France) or water were added to 8 μl of the dilution. The samples were incubated for 60 min at 37°C. The reactions were stopped by adding 1 μl 20 mM EGTA and heating for 10 min at 65°C. 1 μl were employed in 30 μl PCR reactions using GoTaq polymerase (Promega, Charbonnières-les-Bains, France) and forward (GATTCCCACACACTTGTGGCTG) and reverse (TACATGCGGCCGCACGCGTCAGCTGCTGCTCTTGCC) primers encompassing the GFP11 sequence at 200 nM concentration. Cycling conditions were 2 min initial denaturation at 95°C, followed by 30–33 cycles (30 s denaturation at 95°C, 30 s annealing at 52°C, 50 s prolongation at 72) and final prolongation at 72°C for 5 min. To test efficiency of the DNase digestion, 8 μl tenfold diluted leaf extract from a mock-inoculated turnip plant was spiked with 30 ng of pGreen 35S B-JI plasmid DNA and treated as indicated above. For sequencing and deletion analysis, the DNase step was omitted and PCR samples were used directly in Sanger sequencing. For sequencing the entire P6 ORF, the primer couples GATTCCCACACACTTGTGGCTG/GAGGCATCTTGAACGATAGC and GGCGAACAGTTCATACAGAG/GGTGAGGTTTTACCCTCTTGAG, and for P2 analysis the primer couple TGACCATAACCTATATCGTAGG/CTTTAGGCTGATTGCCTAAGGC were used.

## Supporting information

S1 Fig Plant symptoms. (PDF)

S2 Fig Deletion of ORF P2. (PDF)

S3 Fig Detection of encapsidated DNA. (PDF)

S1 Data Raw data. (ZIP)
