# Supplementary material for: Split green fluorescent protein as a tool to study infection with a plant pathogen, Cauliflower mosaic virus
Source: PLoS One. 2019 Mar 6;14(3):e0213087. doi: 10.1371/journal.pone.0213087 (PMC6402836; doi:10.1371/journal.pone.0213087)
Supplement: S1 Fig — (PDF) [file pone.0213087.s001.pdf]

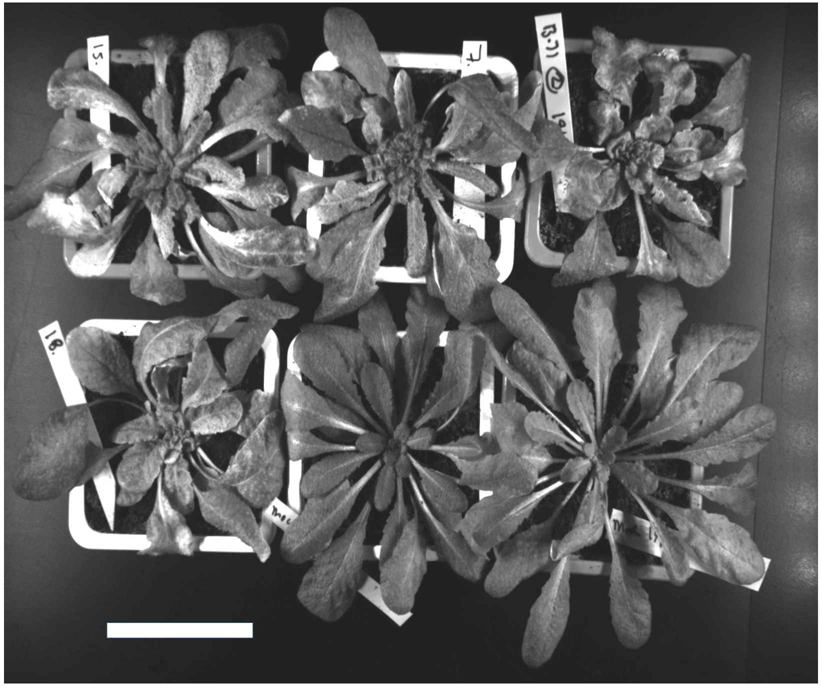

S1 Fig. Symptoms of CaMV$_{wt}$ and CaMV$_{11P6}$ in *A. thaliana* Col0 and transgenic *GFP1-10* plants. Plants were inoculated mechanically with infected plant sap and observed at 31 dpi. Scale bar 5 cm.
