# Supplementary material for: Split green fluorescent protein as a tool to study infection with a plant pathogen, Cauliflower mosaic virus
Source: PLoS One. 2019 Mar 6;14(3):e0213087. doi: 10.1371/journal.pone.0213087 (PMC6402836; doi:10.1371/journal.pone.0213087)
Supplement: S2 Fig — (PDF) [file pone.0213087.s002.pdf]

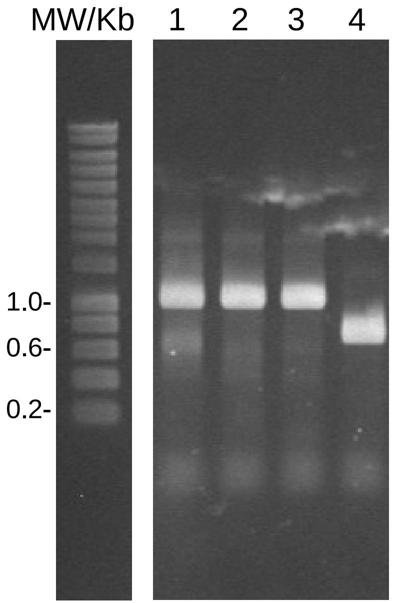

S2 Fig. PCR analysis of P2 ORF. The genomic region encompassing the P2 ORF was amplified by PCR from total extracts prepared from plants infected with $CaMV_{wt}$ (1) or $CaMV_{11P6}$ (2-4) as indicated. Amplification of the whole P2 sequence yielded a 1044 bp product.
