# Supplementary material for: Split green fluorescent protein as a tool to study infection with a plant pathogen, Cauliflower mosaic virus
Source: PLoS One. 2019 Mar 6;14(3):e0213087. doi: 10.1371/journal.pone.0213087 (PMC6402836; doi:10.1371/journal.pone.0213087)
Supplement: S3 Fig — (PDF) [file pone.0213087.s003.pdf]

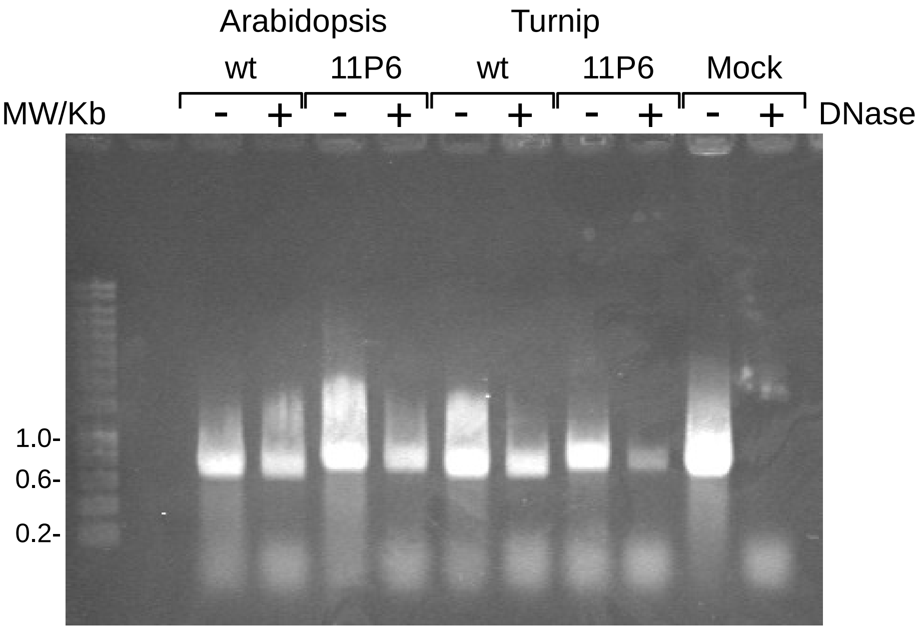

S3 Fig. Detection of encapsidated CaMV DNA by DNase resistance assay. The genomic region encompassing the 11GFP tag was amplified by PCR from total extracts prepared from plants infected with $CaMV_{wt}$ or $CaMV_{11P6}$ as indicated. Before PCR, extracts were (+) or were not (-) incubated with DNase to digest free DNA. To verify efficiency of the DNase treatment, extracts from mock-inoculated turnip leaf were spiked with CaMV encoding plasmid DNA before DNase treatment (Mock). Amplification of $CaMV_{w}$ DNA yielded a 655 bp product, amplification of $CaMV_{11P6}$ DNA a 721 bp product.
