# Supplementary figures and images for: Split green fluorescent protein as a tool to study infection with a plant pathogen, Cauliflower mosaic virus
Source: PLoS One. 2019 Mar 6;14(3):e0213087. doi: 10.1371/journal.pone.0213087 (PMC6402836; doi:10.1371/journal.pone.0213087)

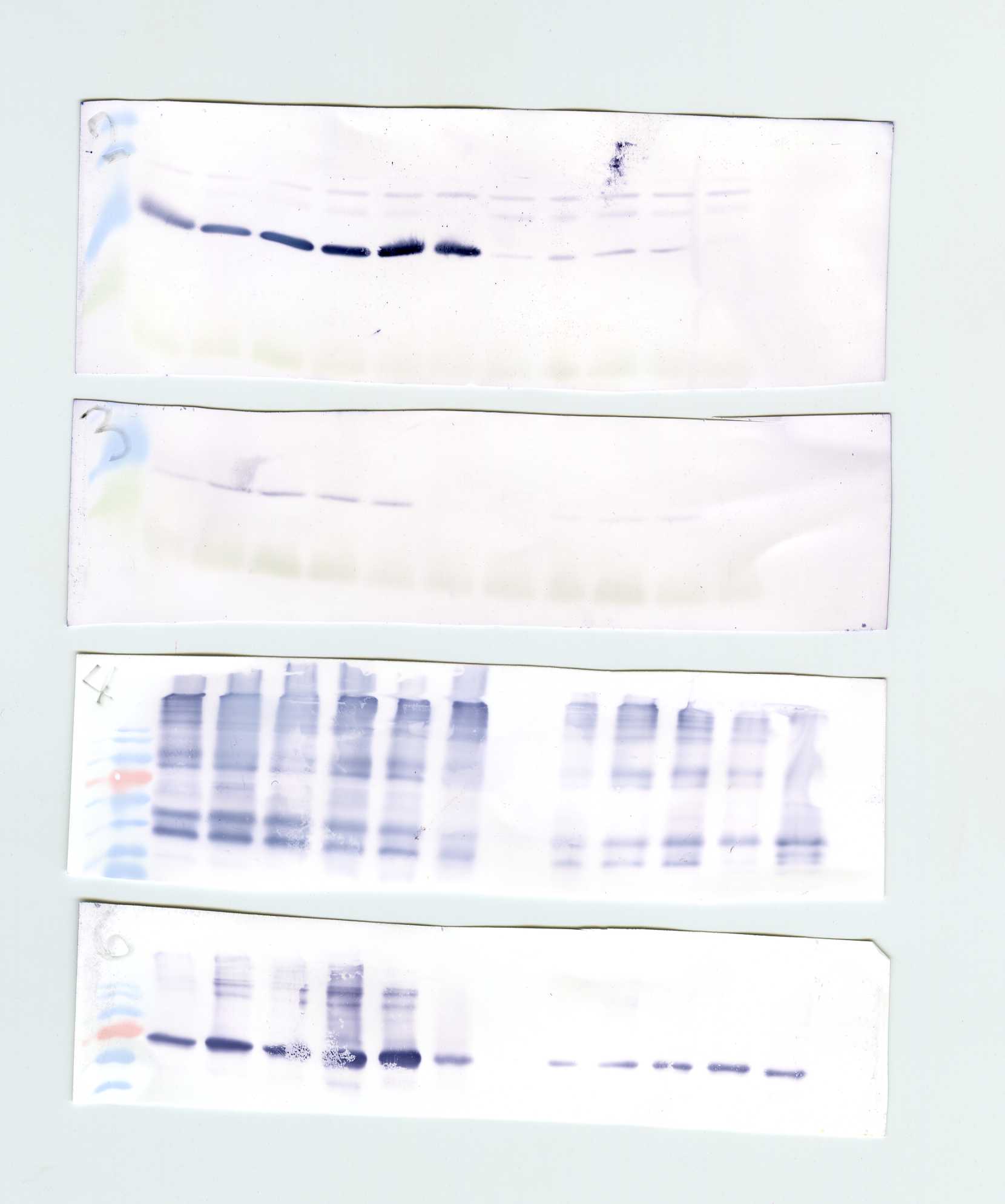

Supplement: S1 Data — (ZIP) [file pone.0213087.s004.zip › Figure3/cinétique protéines révélation phosphatase alcaline027.jpg]

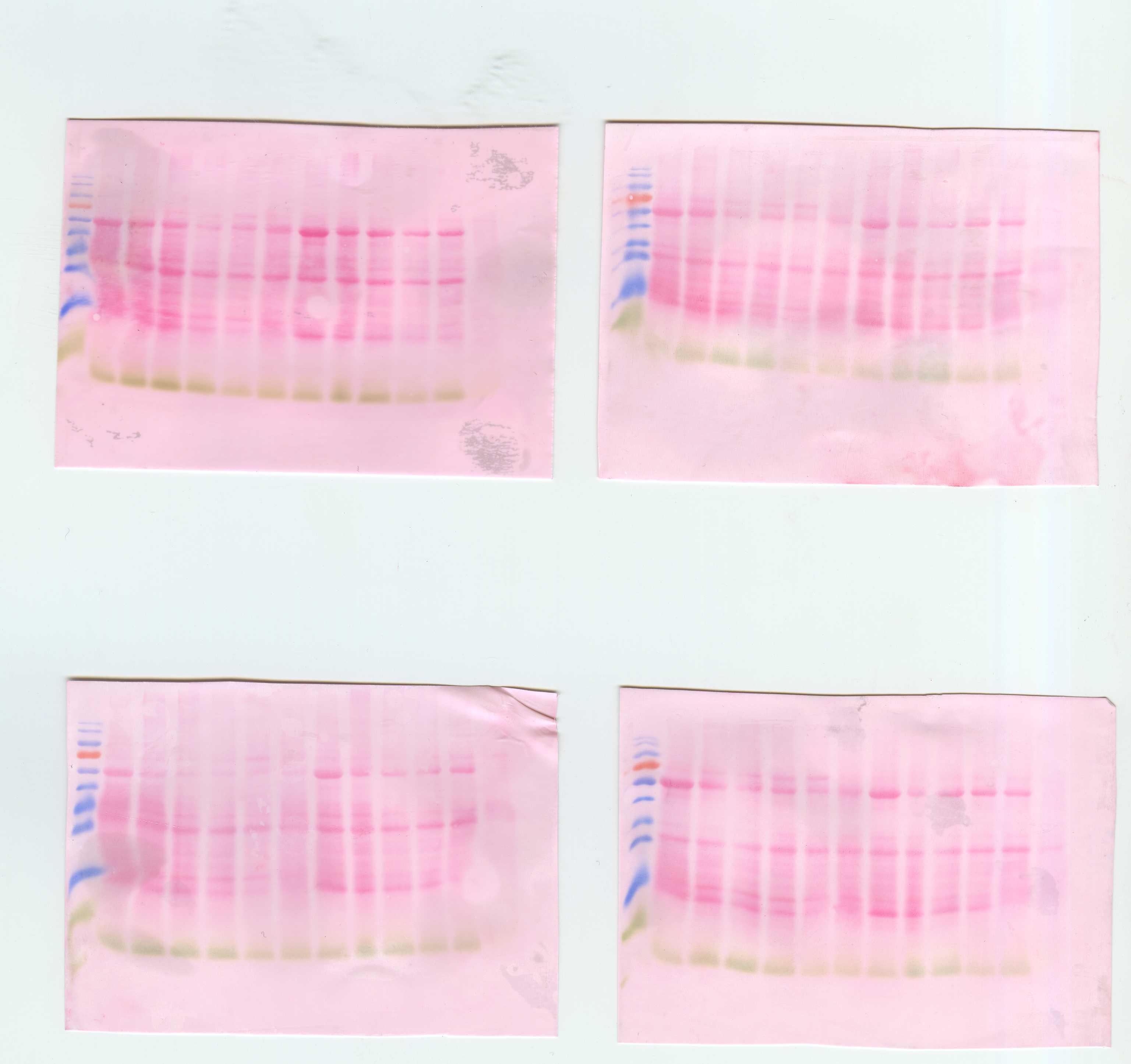

Supplement: S1 Data — (ZIP) [file pone.0213087.s004.zip › Figure3/Ponceauwt10 mut6 cinétique 18.12.2018.jpg]

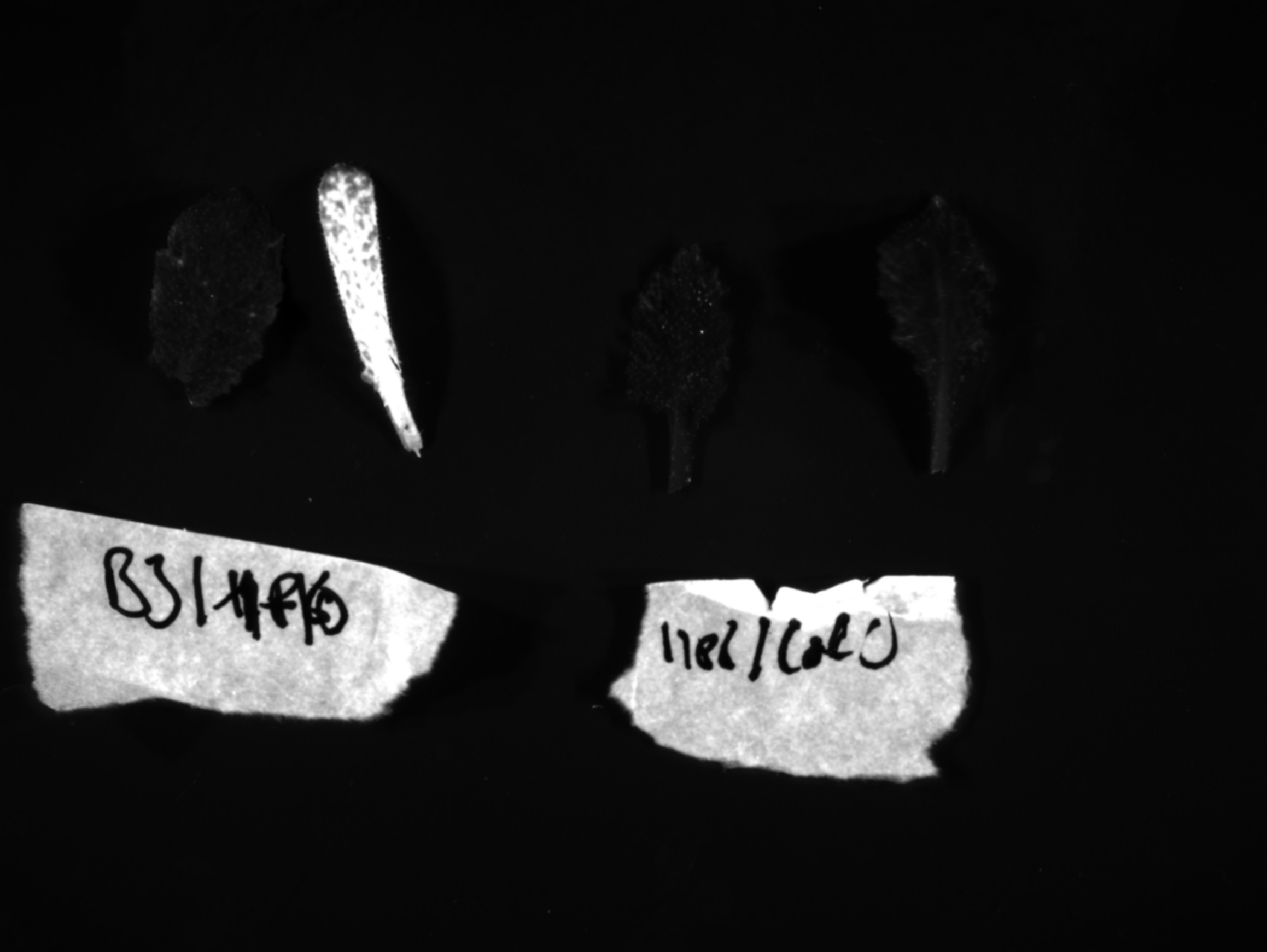

Supplement: S1 Data — (ZIP) [file pone.0213087.s004.zip › Figure4/32dpi01.jpg]

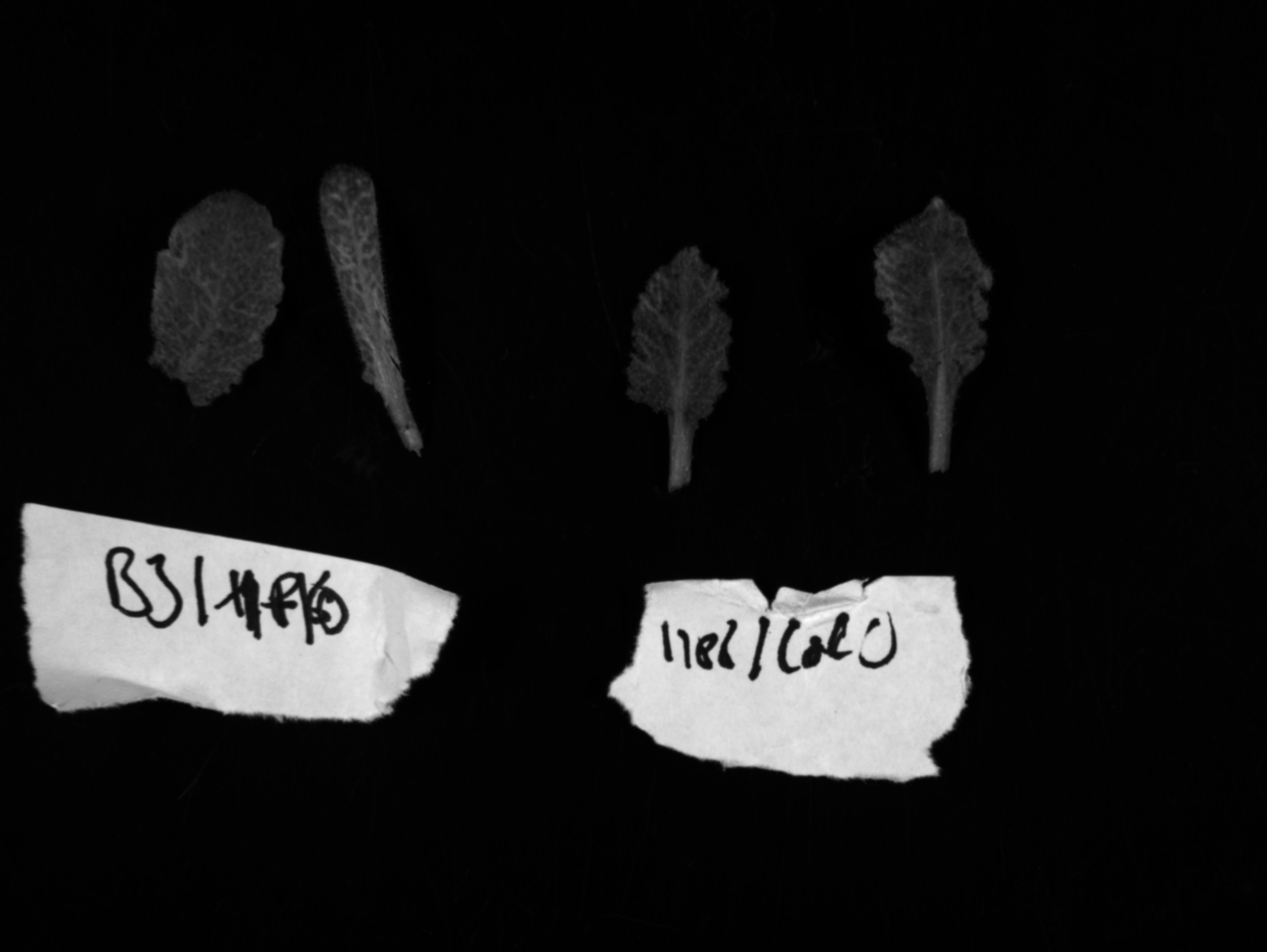

Supplement: S1 Data — (ZIP) [file pone.0213087.s004.zip › Figure4/32dpi02.jpg]

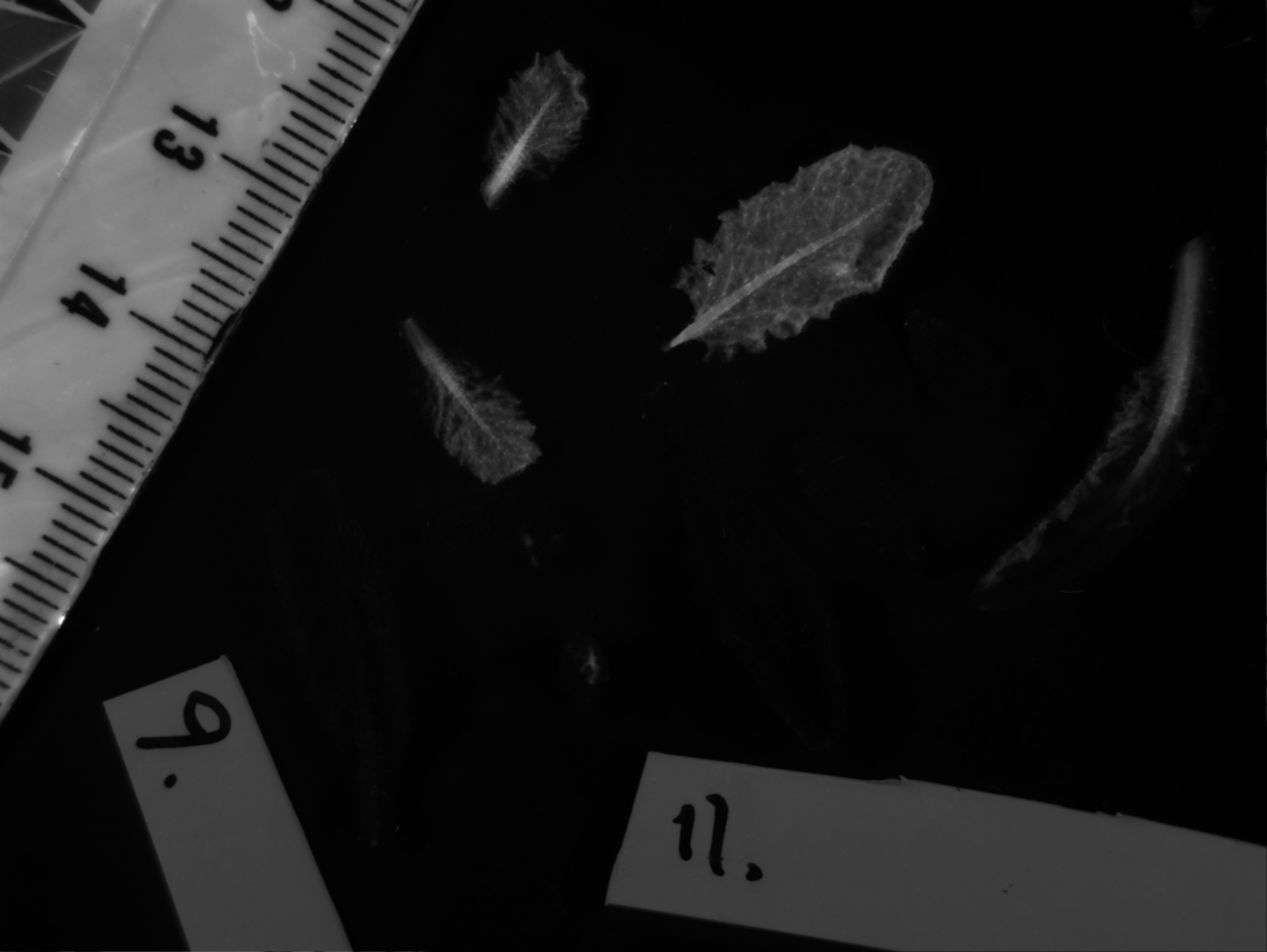

Supplement: S1 Data — (ZIP) [file pone.0213087.s004.zip › Figure4/Plante11GFP-11P6.Plante9col0-11P6-24dpi-short01.jpg]

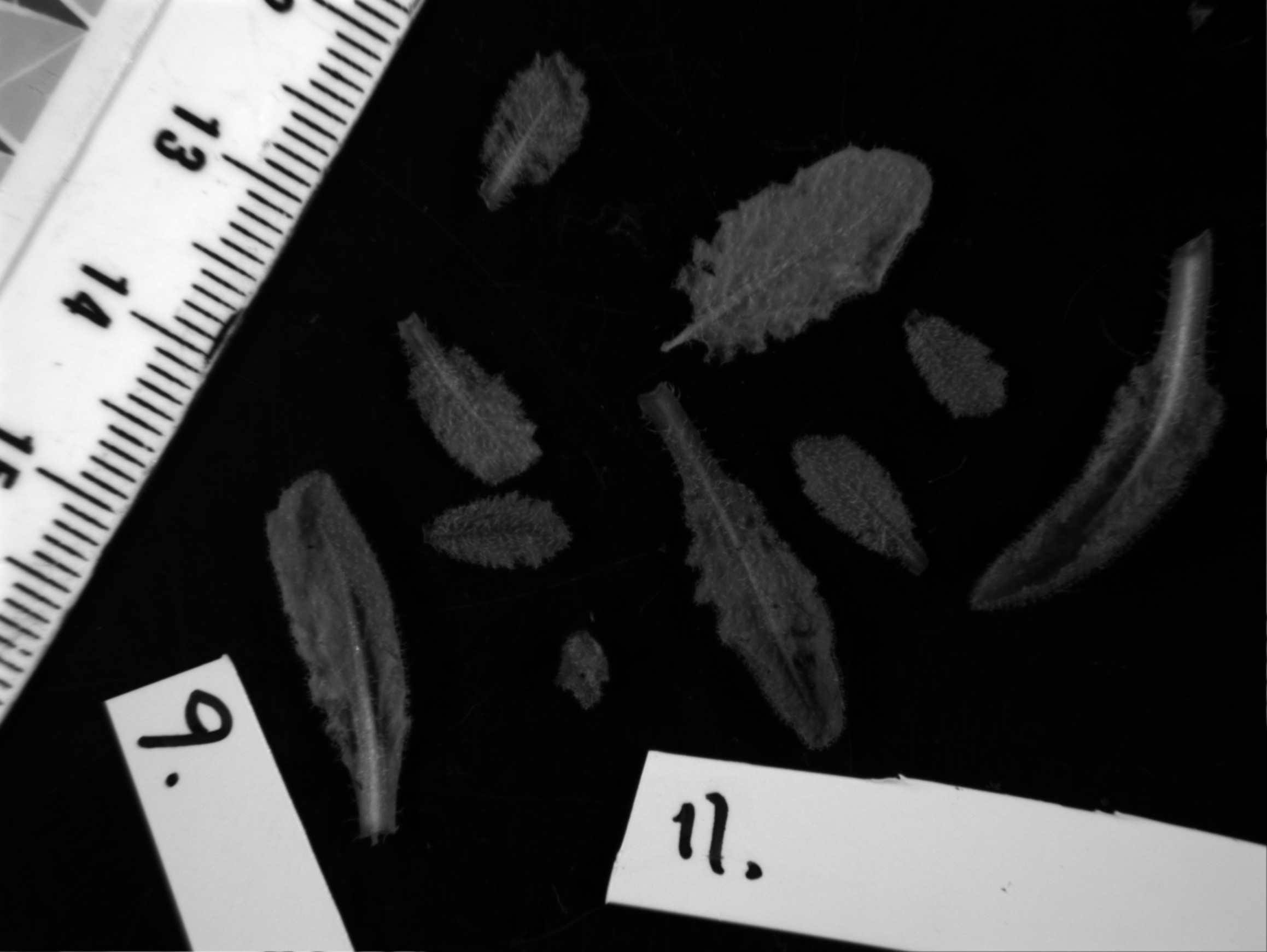

Supplement: S1 Data — (ZIP) [file pone.0213087.s004.zip › Figure4/Plante11GFP-11P6.Plante9col0-11P6-24dpi-short02.jpg]

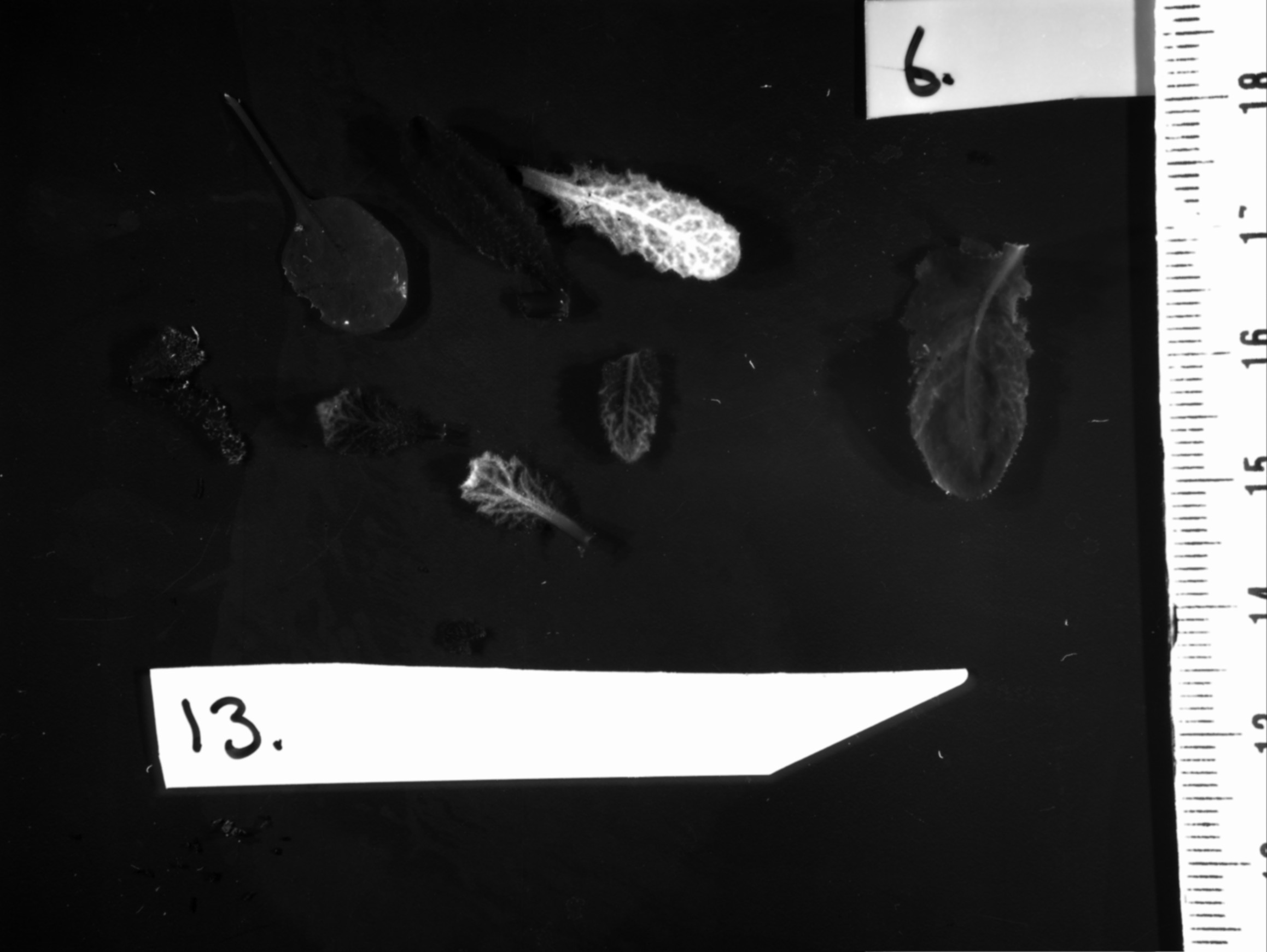

Supplement: S1 Data — (ZIP) [file pone.0213087.s004.zip › Figure4/Plante13GFP-11P6.Plante6GFP-BJI-3min_24dpi01.jpg]

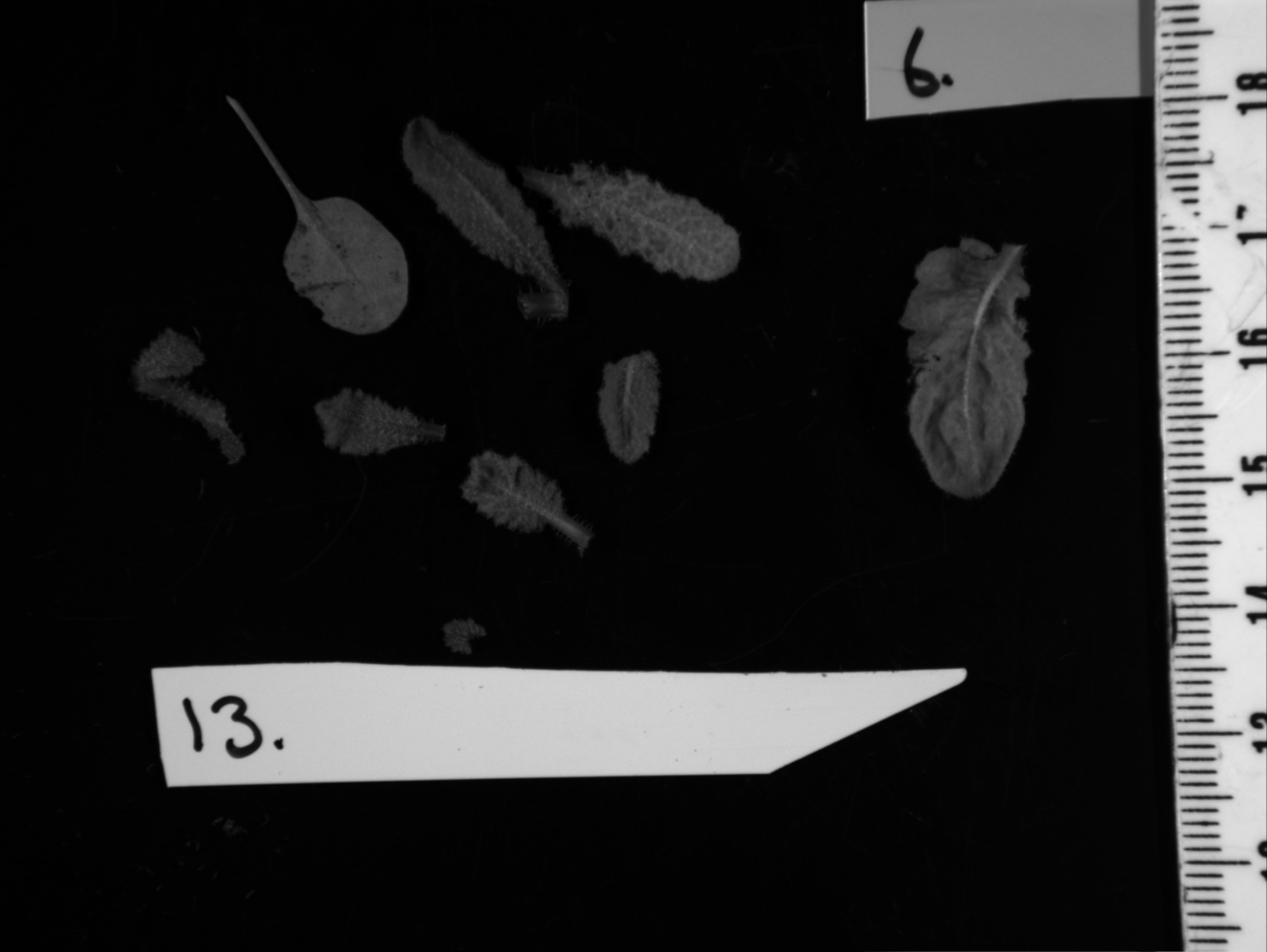

Supplement: S1 Data — (ZIP) [file pone.0213087.s004.zip › Figure4/Plante13GFP-11P6.Plante6GFP-BJI-3min_24dpi02.jpg]

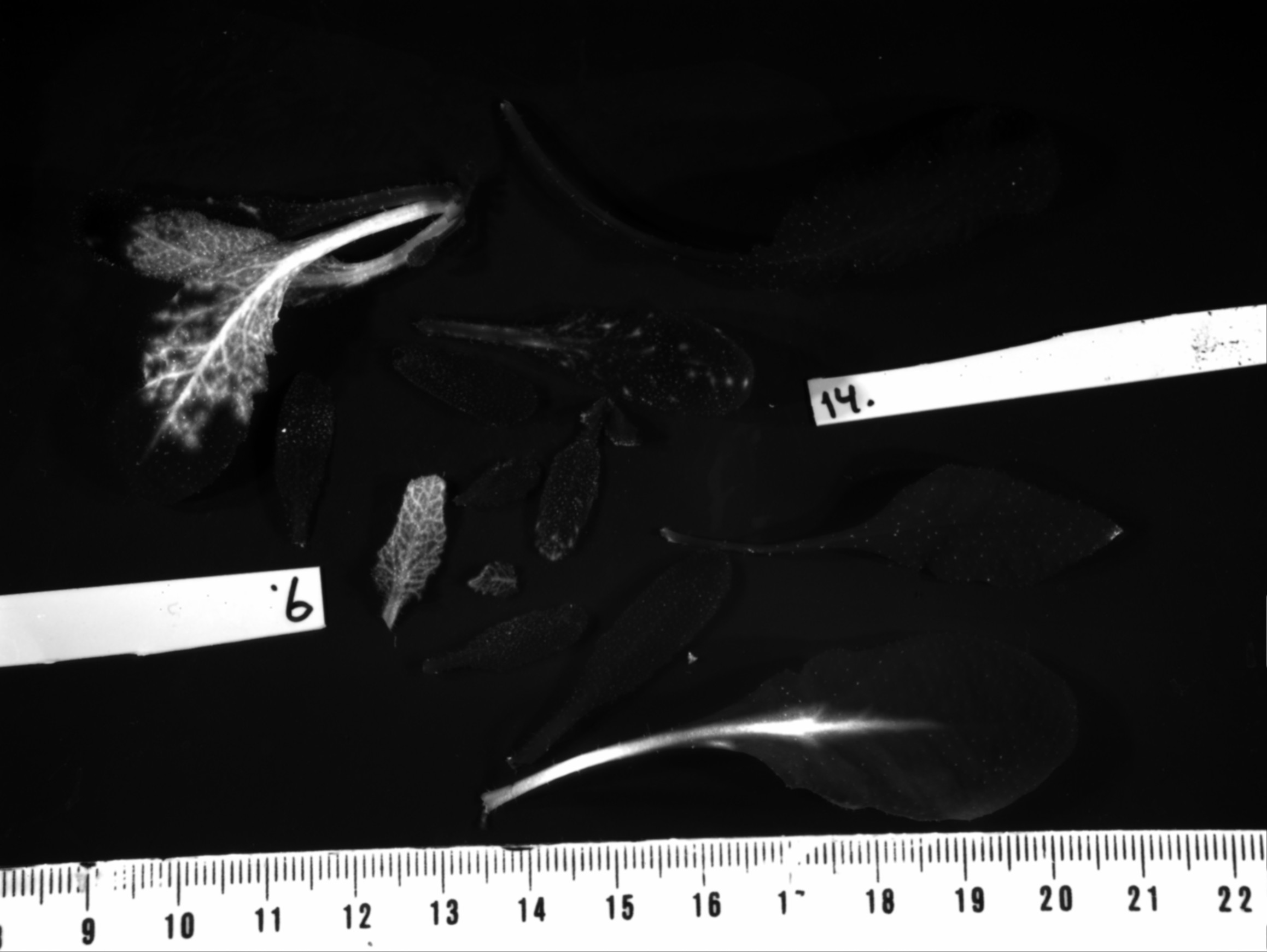

Supplement: S1 Data — (ZIP) [file pone.0213087.s004.zip › Figure4/Plante14GFP-11P6-Plante9Col0-11P6_24dpi401.jpg]

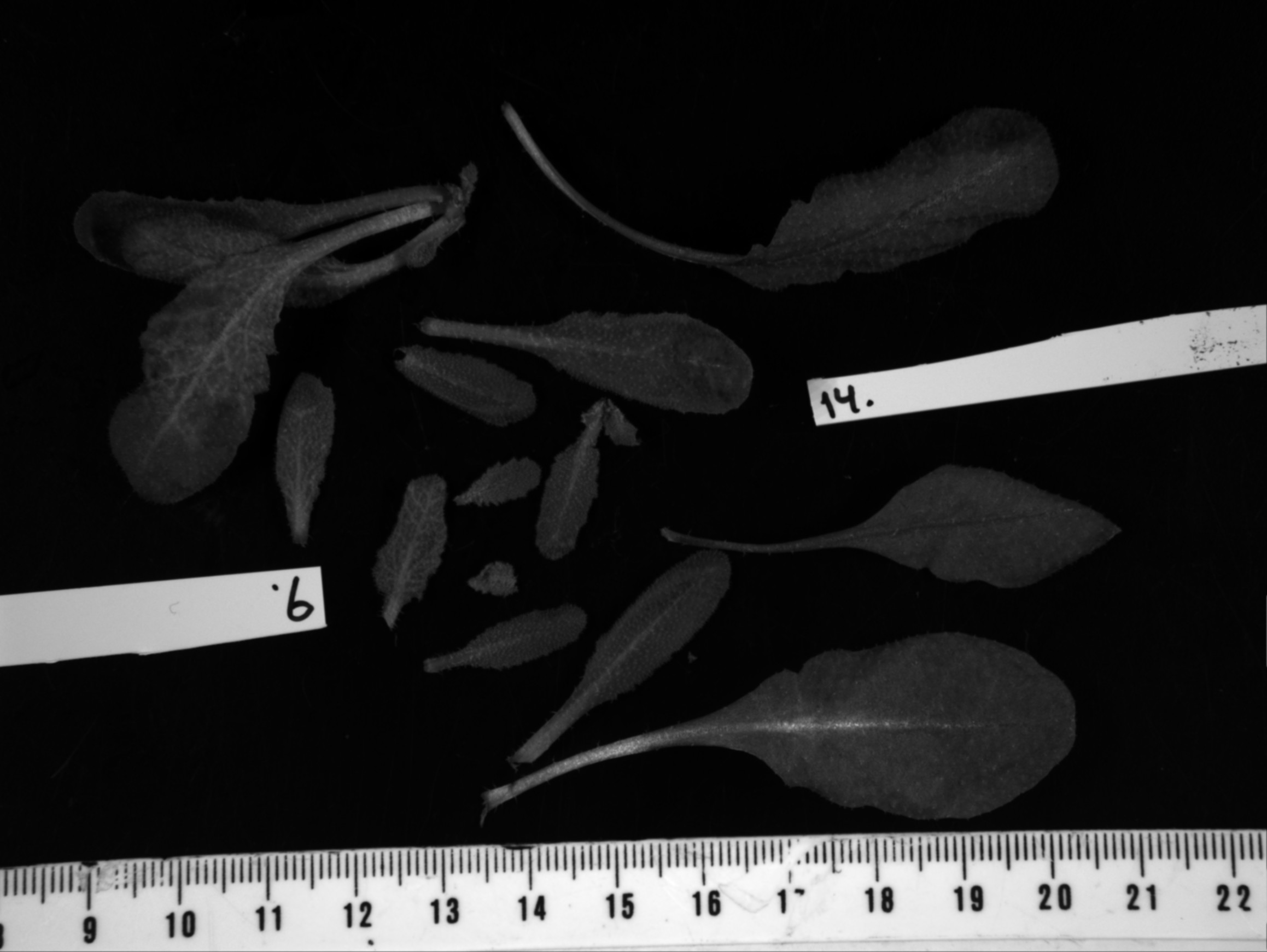

Supplement: S1 Data — (ZIP) [file pone.0213087.s004.zip › Figure4/Plante14GFP-11P6-Plante9Col0-11P6_24dpi402.jpg]

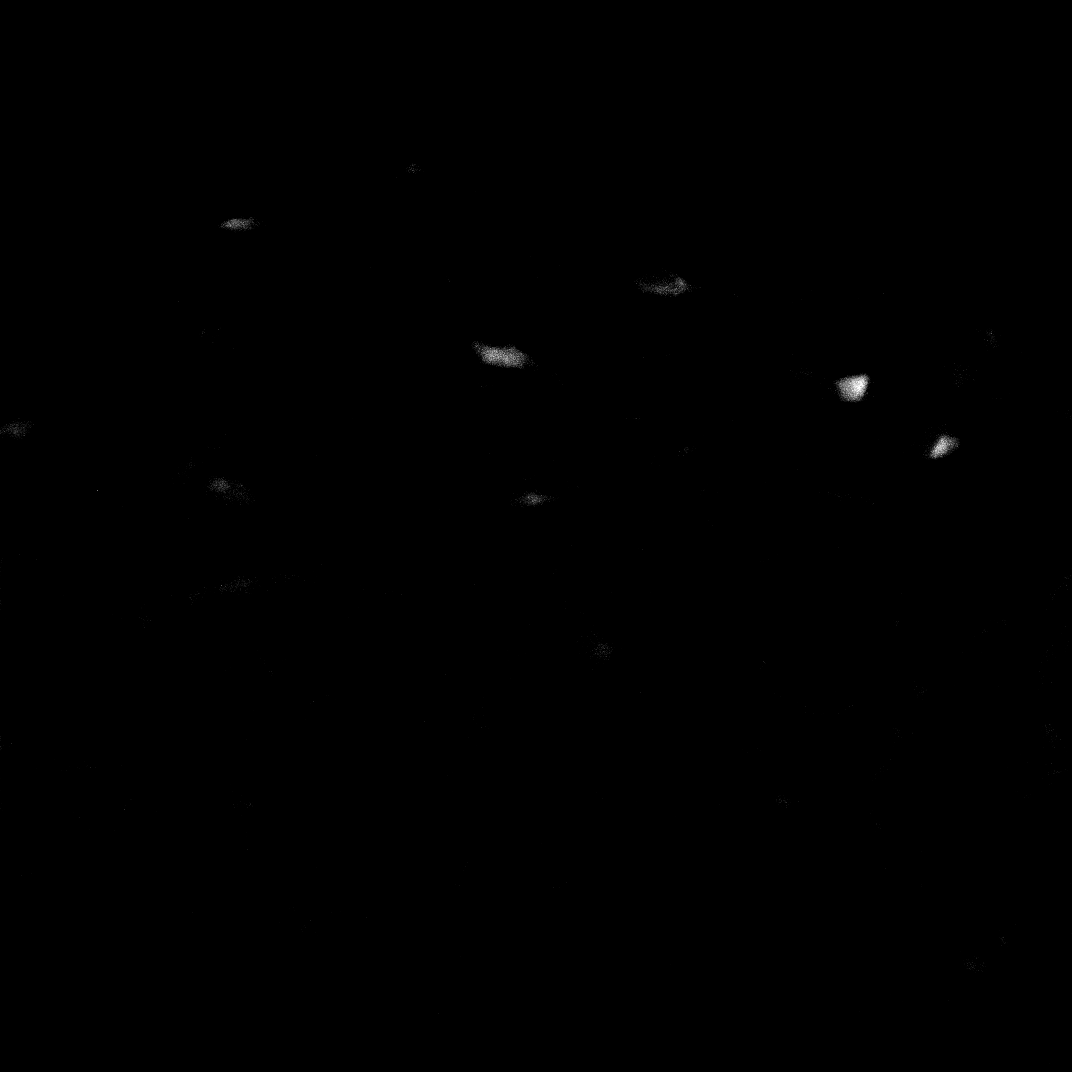

Supplement: S1 Data — (ZIP) [file pone.0213087.s004.zip › Figure5/3dpi_GFPP6-Plant7-5_Maximumintensityprojection.tif]

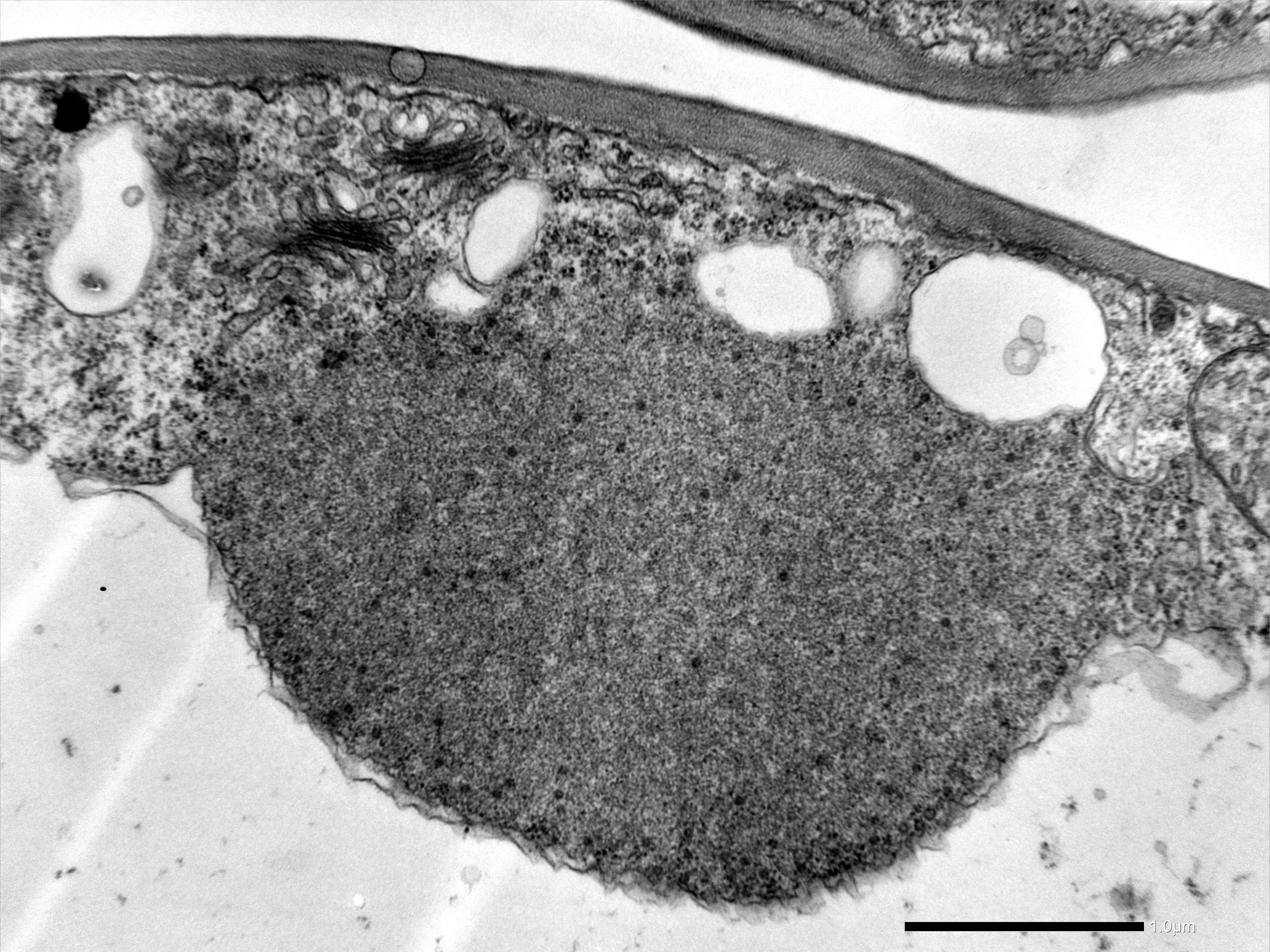

Supplement: S1 Data — (ZIP) [file pone.0213087.s004.zip › Figure5/410_2017- Grille 01.jpg]

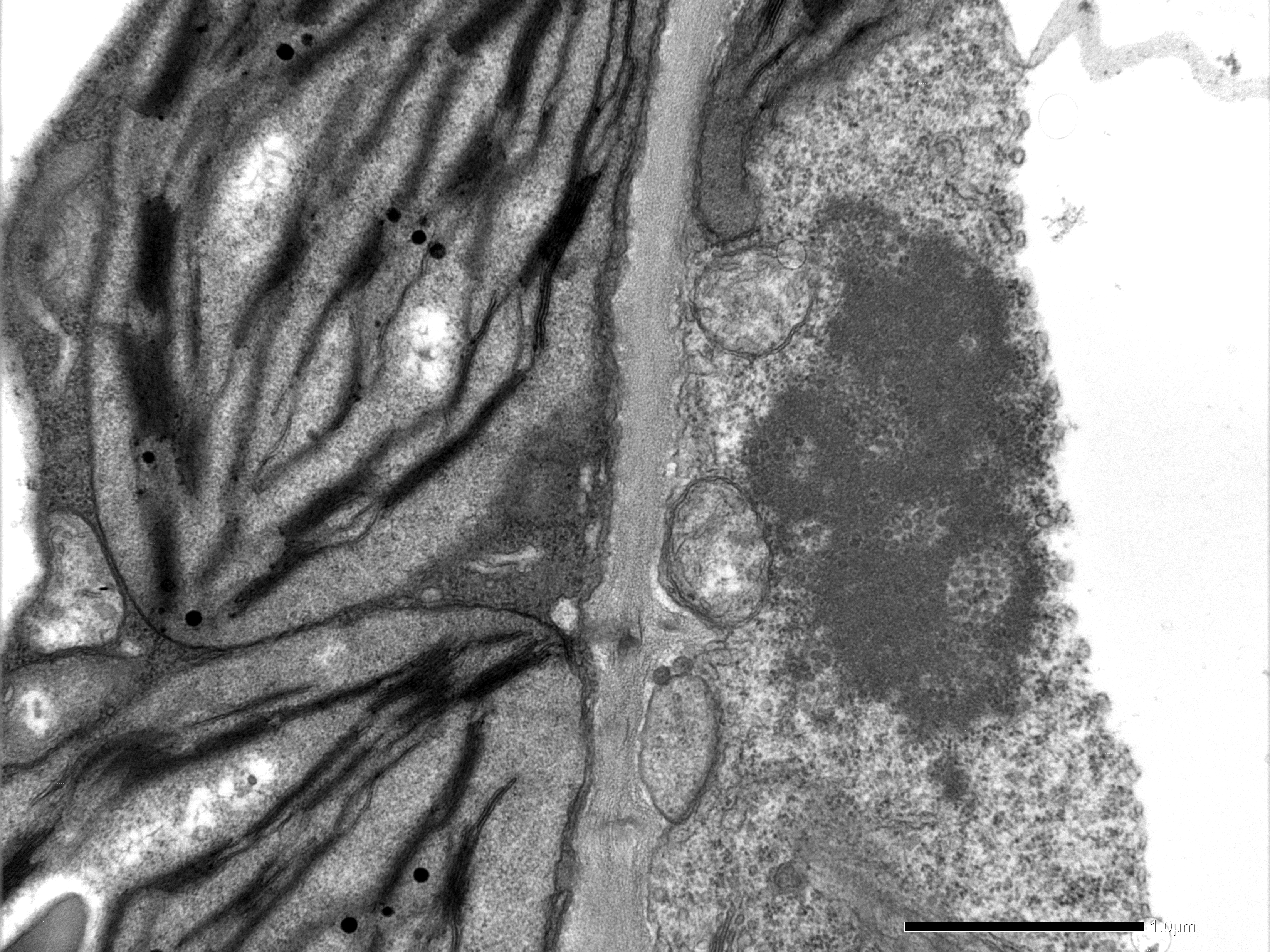

Supplement: S1 Data — (ZIP) [file pone.0213087.s004.zip › Figure5/413_2017- Grille 01.jpg]

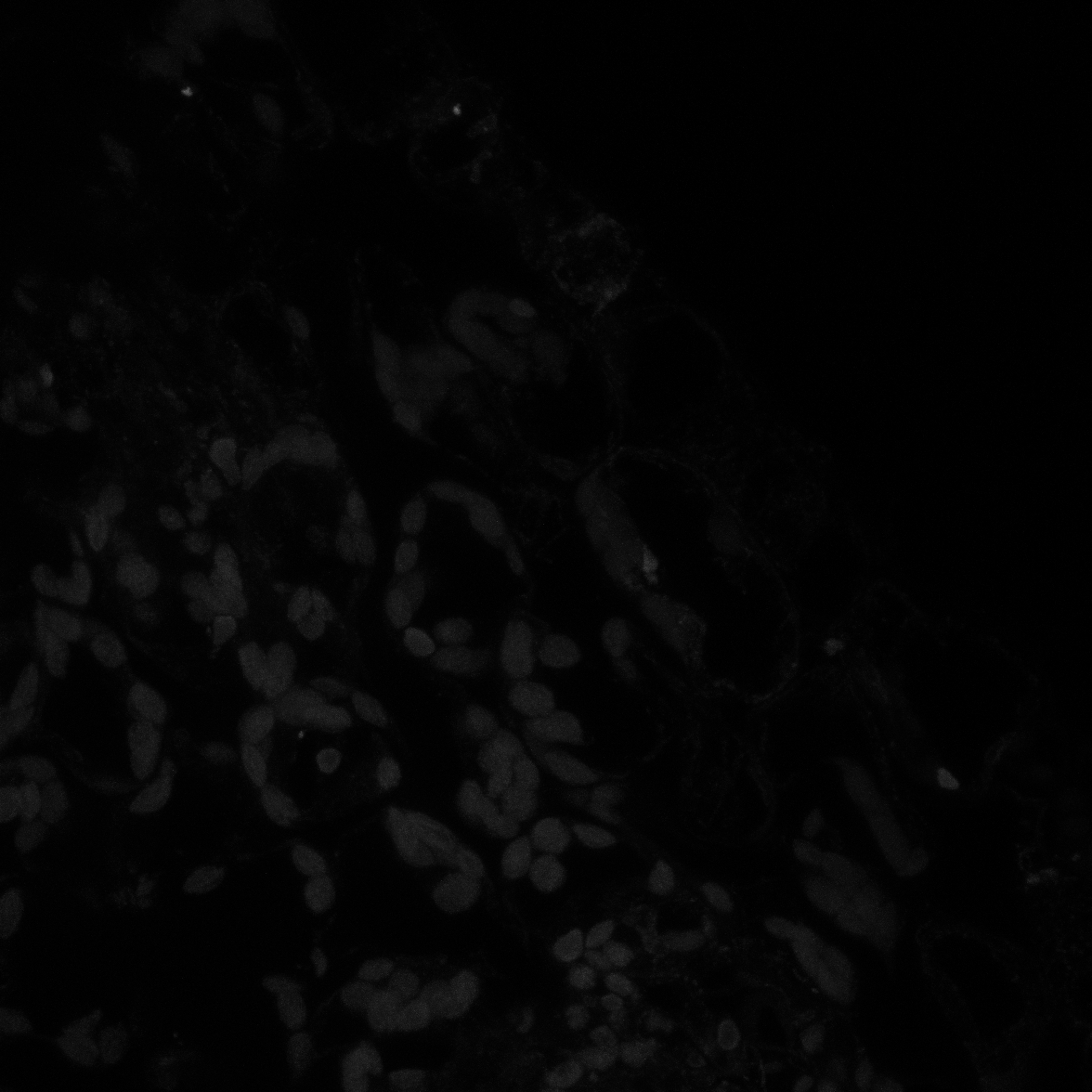

Supplement: S1 Data — (ZIP) [file pone.0213087.s004.zip › Figure5/Col0-P2r-tem-02_Maximumintensityprojection.tif]

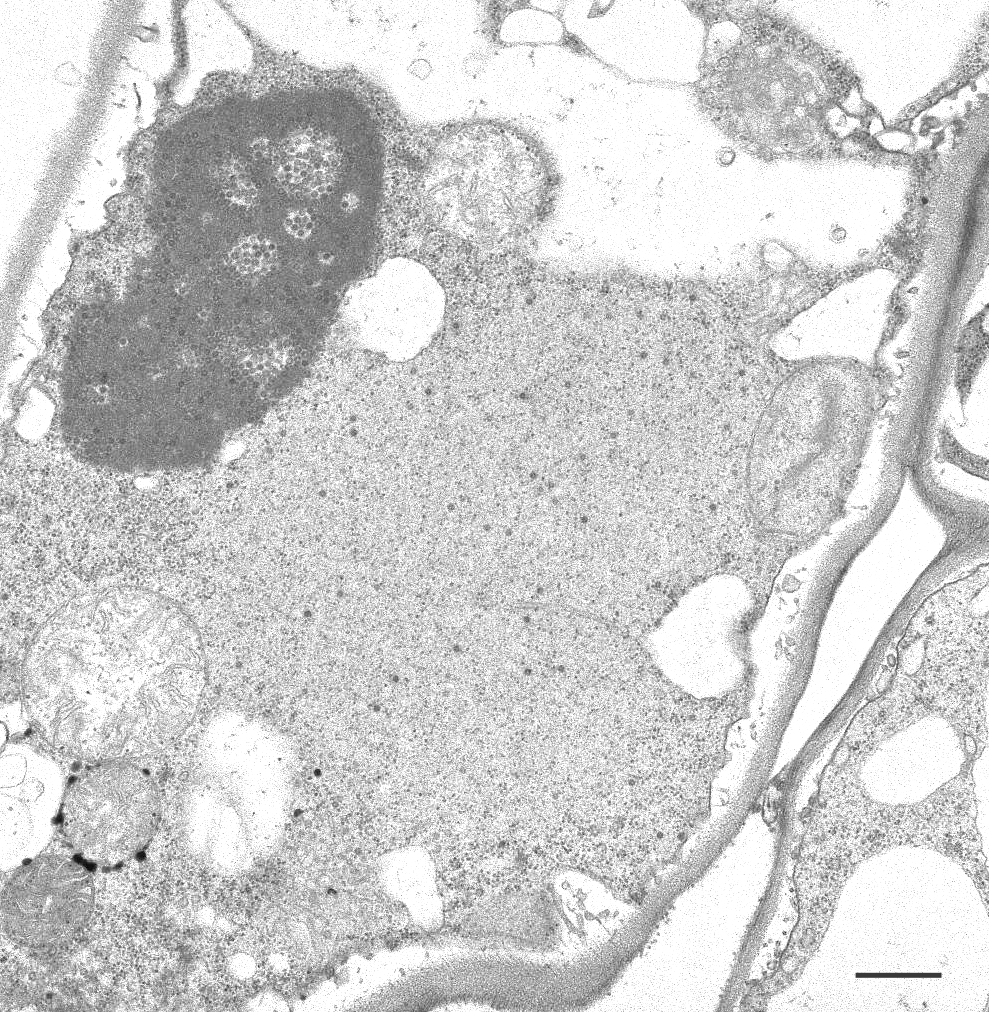

Supplement: S1 Data — (ZIP) [file pone.0213087.s004.zip › Figure5/Usines-CT-CaMV.tif]

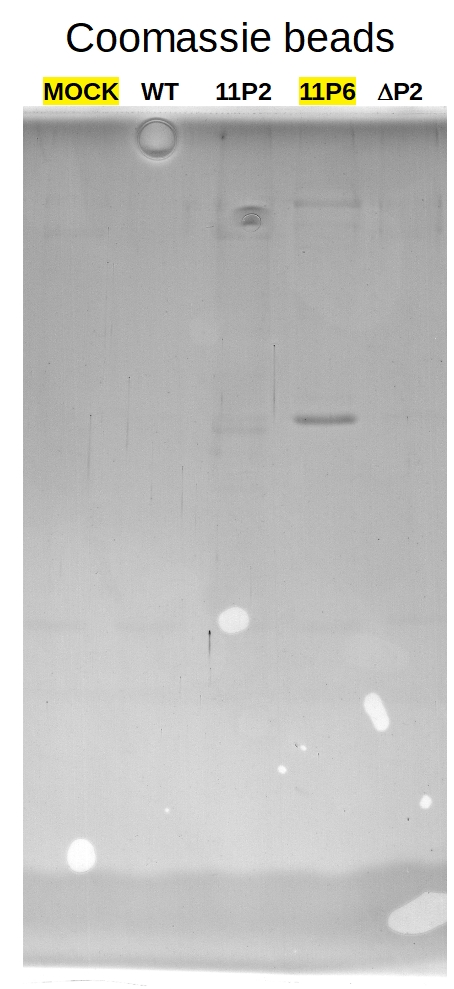

Supplement: S1 Data — (ZIP) [file pone.0213087.s004.zip › Figure7/Coomassie-beads.jpg]

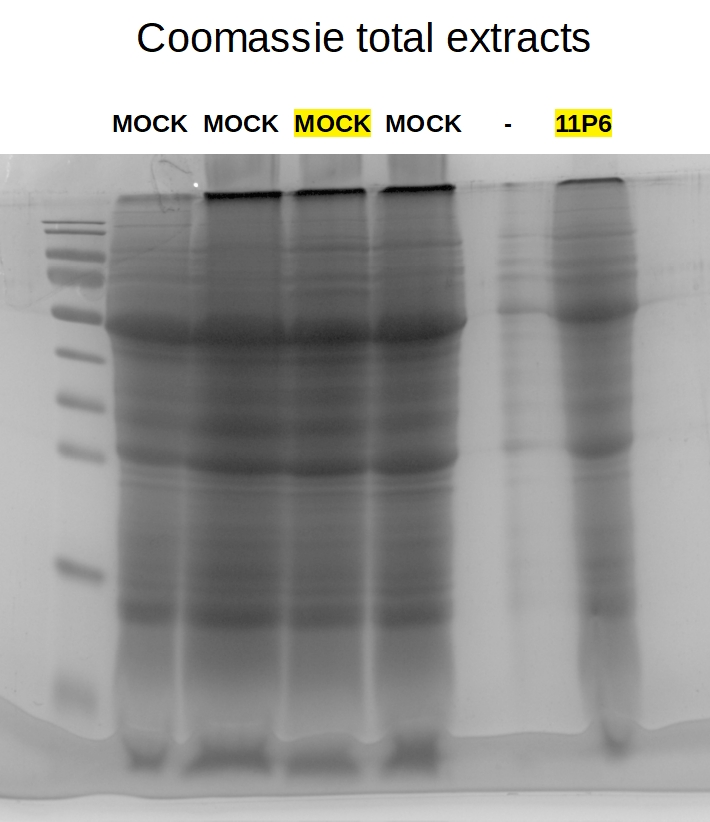

Supplement: S1 Data — (ZIP) [file pone.0213087.s004.zip › Figure7/Coomassie-total.jpg]

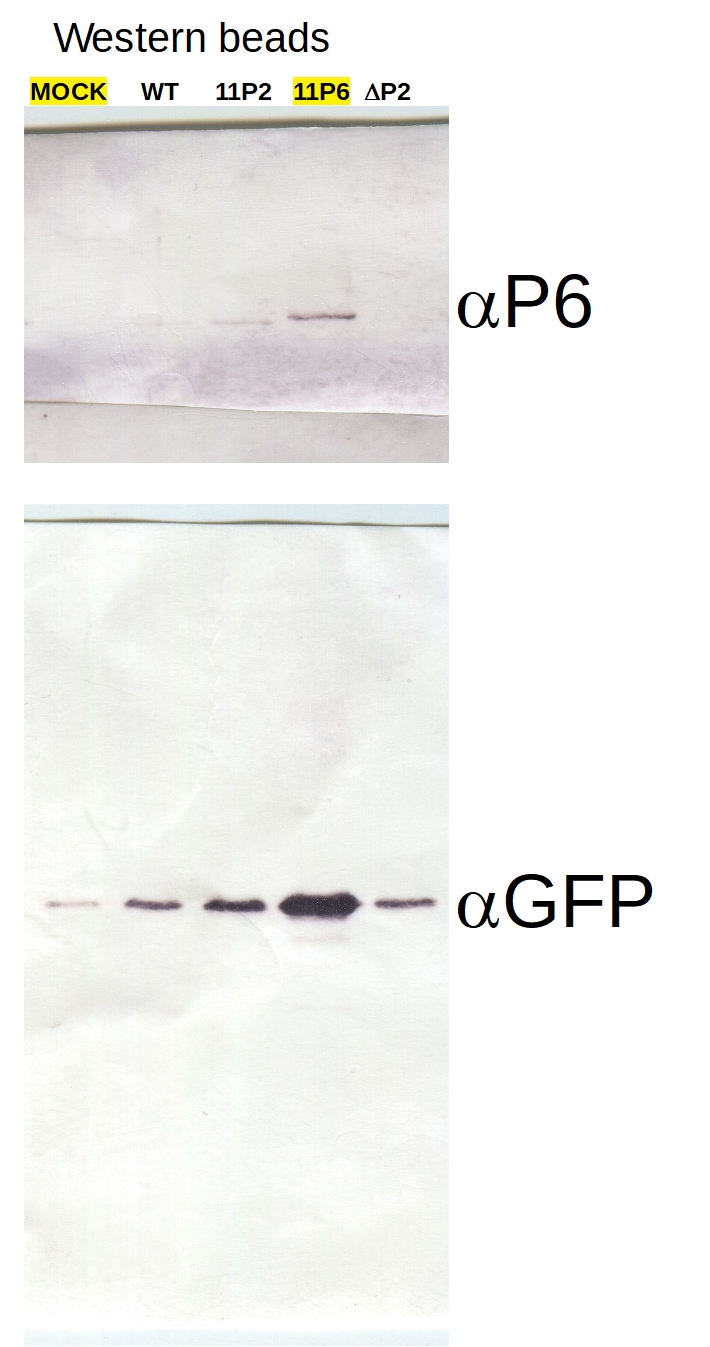

Supplement: S1 Data — (ZIP) [file pone.0213087.s004.zip › Figure7/Western-beads.jpg]

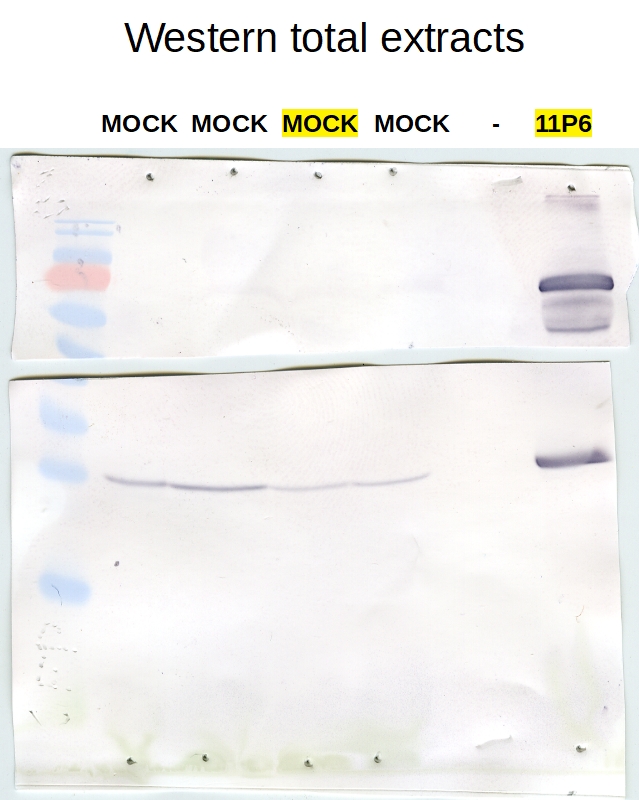

Supplement: S1 Data — (ZIP) [file pone.0213087.s004.zip › Figure7/Western-total.jpg]
