# Supplementary material for: Split green fluorescent protein as a tool to study infection with a plant pathogen, Cauliflower mosaic virus
Source: PLoS One. 2019 Mar 6;14(3):e0213087. doi: 10.1371/journal.pone.0213087 (PMC6402836; doi:10.1371/journal.pone.0213087)
Supplement: S1 Data — (ZIP) [file pone.0213087.s004.zip › Sequencing-results.doc]

Sequencing Arabidopsis from 17 November 2016

>ARAB-P6_MLU6321NOT-R_14 status=passed nucl=692 crlStart=46 crlStop=692 crlLen=647 order=COL16-0CQI
acggactgtgtatagtgacaatttccatgagtgcactccgggagcGCCGG
TAAGCAGCCGCCTTTGTACAACCCCAGTCATCGTATATACCGGCATGTGG
ACCGTTATACACAACGTAGTAGTTGATATGAGGGTGTTGAATACCCGATT
CTGCTCTGAGAGGAGCAACTGTGCTGTTAAGCTCAGATTTTTGTGGGATT
GGAATTCCCTGATGAGGACGTAAAAAGTCCTCTGGCACTGCAAGCCTTAT
CCCAGTTTGAACTGGGTTCATATCTTTTGGCAAGATATTAGCCATCAACG
GATTTGTTGATTCTTTACCAGGAGCCGTTTGCTCTGGAATAGCCTTTACT
TGAGTTGGCGTAAAAGTGGCCAGTGCAGAATGAACTGCTTCTTCTTTTAC
CGGTGTTTCACGGTGGAGAGGGAGGTCTCCTTGTTGCGAAGAGCCGTTAG
CTCTTGCTAAGCTTATTTTTGCTCTTACTAGATCGAGTTCTAGCATTAGT
ATTTTCTCTTGCATGAGGAGTTTTTCTATGTTCTCGCTGCCGCCGCCGCC
GTCTGTGATGCCGGCGGCGTTCACGTACTCGTGCAGCACCATGTGGTCCC
GCATGCTTGGAGGTCTGATTTTCTCAGTCTCCAGAGATGTGTTTATATAG
GCAGTAGCCTTTTGATATCAGCCACAAGTGTGTTGGGAATCA

Sequencing turnip from 17 November 2016

>NAVET-P6-2_MLU6321NOT-R_2 status=passed nucl=688 crlStart=5 crlStop=676 crlLen=672 order=COL16-0CQI
gggtCTGTATAGTGGCAATTTCTTGTGTGCAACTCCGGGAACGCCGTTTG
TTGCCGCCTTTGTACAACCCCAGTCATCGTATATACCGGCATGTGGACCG
TTATACACAACGTAGTAGTTGATATGAGGGTGTTGAATACCCGATTCTGC
TCTGAGAGGAGCAACTGTGCTGTTAAGCTCAAATTTTTGTGGGATTGGAA
TTCCCTGATGAGGACGTAAAAAGTCCTCTGGCACTGCAAGCCTTATCCCA
GTTTGAACTGGGTTCATATCTTTTGGCAAGATATTAGCCATCAACGGATT
TGTTGATTCTTTACCAGGAGCCGTTTGCTCTGGAATAGCCTTTACTTGAG
TTGGCGTAAAAGTGGCCAGTGCAAAATGAACTGCTTCTTCTTTTACCGGT
GTTTCACGGTGGAGAGGGAGGTCTCCTTGTTGCGAAGAGCCGTTAGCTCT
TGCTAAGCTTATTTTTGCTCTTACTAGATCGAGTTCTAGCATTAGTATTT
TCTCTTGCATGAGGAGTTTTTCTATGTTCTCGCTGCCGCCGCCGCCGTCT
GTGATGCCGGCGGCGTTCACGTACTCGTGCAGCACCATGTGGTCCCGCAT
GCTTGGAGGTCTGATTTTCTCAGTCTCCAGAGATGTGTTTATATAGGCAG
TATCCTTTTGATATCAGCCACGAGTGtggggagaaatc

Sequencing Arabidopsis from 18 May 2018

>DRUCKER_C6-1014_B02_014.ab1
NNNNNNNNNNNNNNNNNNNNNNNNNNTCTCTGGAGACTGAGAAAATCAGACCTCCNAGCATGCGGGACCACATGGTGCTG
CACGAGTACGTGAACGCCGCCGGCATCACAGACGGCGGCGGCGGCAGCGAGAACATAGAAAAACTCCTCATGCAAGAGAA
AATACTAATGCTAGAACTCGATCTAGTAAGAGCAAAAATAAGCTTAGCAAGAGCTAACGGCTCTTCGCAACAAGGAGACC
TCCCTCTCCACCGTGAAACACCGGTAAAAGAAGAAGCAGTTCATTCTGCACTGGCCACTTTTACGCCAACTCAAGTAAAG
GCTATTCCAGAGCAAACGGCTCCNGGTAAAGAATCAACAAATCCGTTGATGGCTAATATCTTGCCAAAAGATATGAACCC
AGTTCAAACTGGGATAAGGCTTGCAGTGCCAGAGGACTTTTTACGTCCTCATCAGGGAATTCCAATCCCACAAAAATCTG
AGCTTAACAGCACAGTTGCTCCTCTCAGAGCAGAATCGGGTATTCAACACCCTCATATCAACTACTACGTTGTGTATAAC
GGTCCACATGCCGGTATATACGATGACNGGGGTTGTACAAAGGCGGCAACAAACGGCGTTCCCGGAGTTGCACACAAGAA
ATTTGCCACTATNACAGAGGCAAGAGCAGCAGCTGACGCGTACACAACAAGTCAGCAAACAGACAGGTTGAACTTCATCC
CCAAAGGAGAAGCTCAACTCNNGCCCAAGAGCTTTGCTNAGGCCCTAACAAGCCCACCAAAGCAAAAAGCCCACTGGCTC
ANGCTNGGAACCAAAAGGNCCAGCANTGATCCNGCCCCAAAAGAGATCTCCTTTGCCCCGGANANNNAATGGACGATTTC
CTCTATCTNACNATCTAGGANGAAGTTCGAAGGNGAAGGNGACNANNTATGTTCACCACTGANATGAGAAGNNNGCCTCT
TCANTTCANAANAATGCTGACCCACAGANGGNTAGANAGGCCTA

Sequencing Arabidopsis from 21 November 2018

>DRUCKER_J6-1014_H02_002.ab1
NNNNNNNNNNNNNNNNNANNNNTCTCTGGAGACTGAGAAAATCAGACCTCCNAGCATGCGGGACCACATGGTGCTGCACG
AGTACGTGAACGCCGCCGGCATCACAGACGGCGGCGGCGGCAGCGAGAACATAGAAAAACTCCTCATGCAAGAGAAAATA
CTAATGCTAGAACTCGATCTAGTAAGAGCAAAAATAAGCTTAGCAAGAGCTAACGGCTCTTCGCAACAAGGAGACCTCCC
TCTCCACCGTGAAACACCGGTAAAAGAAGAAGCAGTTCATTCTGCACTGGCCACTTTTACGCCAACTCAAGTAAAGGCTA
TTCCANAGCAAACGGCTCCNGGTAAAGAATCAACAAATCCGTTGATGGCTAATATCTTGCCAAAAGATATGAACCCAGTT
CAAACTGGGATAAGGCTTGCAGTGCCAGAGGACTTTTTACGTCCTCATCAGGGAATTCCAATCCCACAAAAATCTGANCT
TAACAGCACAGTTGCTCCTCTCANAGCAGAATCGGGTATTCAACACCCTCATATCAACTACTACGTTGTGTATAACGGTC
CACATGCCGGTATATACGATGACTGGGGTTGTACAAAGGCGGCAACAAACGGCGTTCCCGGAGTTGCACACAAGAAATTT
GCCACTATTACAGAGGCAAGAGCAGCAGCTGACGCGTACACAACAAGTCAGCAAACAGACAGGTTGAACTTCATCCCCAA
AGGAGAAGCTCAACTCANGCCCAAGAGCTTTGCTAAGGNCCTAACAAGCCCACCAAAGCAAAAAGCCCACTGGCTCACGC
TAGGAACCAAAAGGCCCANCAGTGATCCAGCCCCAAAAGAGATCTCCTTTGCCCCGGANANTACAATGGACGATTTCCTC
TATCTTTACGATCTAGG

Sequencing Arabidopsis from 30 November 2018

>DRUCKER_G6-1014_E02_008.ab1
NNNNNNNNNNNNNNNNNNNNNNNNNNNNNTCTCTGGAGACTGAGAAAATCANACCTCNNAGCATGCGGGACCACATGGTG
CTGCACGAGTACGTGAACGCCGCCGGCATCACAGACGGCGGCAGCGAGAACATAGAAAAACTCCTCATGCAAGAGAAAAT
ACTAATGCTAGAACTCGATCTAGTAAGAGCAAAAATAAGCTTAGCAAGAGCTAACGGCTCTTCGCAACAAGGAGACCTCC
CTCTCCACCGTGAAACACCGGTAAAAGAAGAAGCAGTTCATTCTGCACTGGCCACTTTTACGCCAACTCAAGTAAAGGCT
ATTCCAGAGCAAACGGCTCCTGGTAAAGAATCAACAAATCCGTTGATGGCTAATATCTTGCCAAAAGATATGAACCCAGT
TCAAACTGGGATAAGGCTTGCAGTGCCAGAGGACTTTTTACGTCCTCATCAGGGAATTCCAATCCCACAAAAATCTGAGC
TTAACAGCACAGTTGCTCCTCTCAGAGCAGAATCGGGTATTCAACACCCTCATATCAACTACTACGTTGTGTATAACGGT
CCACATGCCGGTATATACNATGACTGGGGTTGTACAAAGGCGGCAACAAACGGCGTTCCCGGAGTTGCACACAAGAAATT
TGCCACTATTACAGAGGCAAGAGCAGCAGCTGACGCGTACACAACAAGTCAGCAAACAGACAGGTTGAACTTCATCCCCA
AAGGAGAAGCTCAACTCAAGCCCAANAGCTTTGCTAAGGNCCTAACAAGCCCACCAAAGCAAAAAGCCNACTGGCTCACG
CTAGGAACCAAAAGGCCCAGCAGNGATCCNNCCCCAAAAGAGATCTCCTTTGNCCCGGANATTACAATGNACGANTTCCT
CTATCTTTACNATCTANGAAGGANNNTCGAANGNGAANGNGANNANNCTATGTNCACCNCTGATAATGANAAGGNTAGCN
NCTTCANTTTCANAAANAATGCTGACCCNCANATG
